# The Warming Climate Aggravates Atmospheric Nitrogen Pollution in Australia

**DOI:** 10.34133/2021/9804583

**Published:** 2021-06-07

**Authors:** Yi Sun, Baojing Gu, Hans J. M. van Grinsven, Stefan Reis, Shu Kee Lam, Xiuying Zhang, Youfan Chen, Feng Zhou, Lin Zhang, Rong Wang, Deli Chen, Jianming Xu

**Affiliations:** ^1^College of Environmental and Resource Sciences, Zhejiang University, Hangzhou 310058, China; ^2^School of Agriculture and Food, The University of Melbourne, Melbourne, Victoria 3010, Australia; ^3^PBL Netherlands Environmental Assessment Agency, PO BOX 30314, 2500 GH The Hague, Netherlands; ^4^UK Centre for Ecology & Hydrology, Bush Estate, Penicuik, Midlothian EH26 0QB, UK; ^5^University of Exeter Medical School, European Centre for Environment and Health, Knowledge Spa, Truro TR1 3HD, UK; ^6^International Institute for Earth System Science, Nanjing University, Nanjing 210023, China; ^7^Laboratory for Climate and Ocean–Atmosphere Studies, Department of Atmospheric and Oceanic Sciences, School of Physics, Peking University, Beijing 100871, China; ^8^College of Urban and Environmental Sciences, Peking University, Beijing 100871, China

## Abstract

Australia is a warm country with well-developed agriculture and a highly urbanized population. How these specific features impact the nitrogen cycle, emissions, and consequently affect environmental and human health is not well understood. Here, we find that the ratio of reactive nitrogen (*N*_*r*_) losses to air over losses to water in Australia is 1.6 as compared to values less than 1.1 in the USA, the European Union, and China. Australian *N*_*r*_ emissions to air increased by more than 70% between 1961 and 2013, from 1.2 Tg N yr^−1^ to 2.1 Tg N yr^−1^. Previous emissions were substantially underestimated mainly due to neglecting the warming climate. The estimated health cost from atmospheric *N*_*r*_ emissions in Australia is 4.6 billion US dollars per year. Emissions of *N*_*r*_ to the environment are closely correlated with economic growth, and reduction of *N*_*r*_ losses to air is a priority for sustainable development in Australia.

## 1. Introduction

The role of global food, fibre, and biofuel production in the disruption of the global nitrogen (N) cycle has attracted much attention, as better quantification of reactive N (*N*_*r*_, all N forms except N_2_) losses to the environment have highlighted their impacts on environmental quality, ecosystems, and human health [1, 2]. With increasing use of mineral fertilizers and fossil fuels, the magnitude of anthropogenic N fluxes contributed to a tripling of total N input to terrestrial ecosystems compared to the preindustrial era [3]. *N*_*r*_ emitted to the atmosphere through both natural and anthropogenic processes contributes to a range of environmental problems, including ambient air pollution (AAP), stratospheric ozone (O_3_) depletion, global warming, acidification and eutrophication of ecosystems, biodiversity loss, and agricultural and horticultural crop damage [4–7].

A comprehensive assessment of N use efficiency and *N*_*r*_ emissions is of importance to design strategies to increase sustainability of Australia's agriculture, as Australia is one of the leading global exporters of wheat, cotton, wool, and beef. From 1961 to 2013, productivity from cropping land in Australia increased fivefold, while N fertilizer consumption increased 36 times [8], indicating risk of substantial *N*_*r*_ losses to the environment and, at the same time, the scope for significant agricultural N efficiency gains. In addition, nearly 100 million cattle and sheep are reared in Australia in 2018 [9], which make Australia the world's largest red meat exporter by value [10] and emit considerable amounts of *N*_*r*_, to the environment through excretion, mainly in the form of NH_3_ and N_2_O. Fossil fuel combustion is another important source of *N*_*r*_ emissions. Increasing NO_x_ emissions in large cities contribute to fine particulate matter (PM_2.5_) pollution and photochemical smog, with increased risks of respiratory and cardio-vascular diseases [11–13]. The N footprint of average Australian was 47 kg N cap^−1^ yr^−1^, ranking the highest among all evaluated countries [14, 15].

Atmospheric emissions of *N*_*r*_ in Australia have been estimated in previous studies [16, 17]. However, almost all these focused on aggregated contributions from major sectors such as agriculture and industry, without detailing contributions from natural sources or estimating how climate trend affects major N fluxes. Also, the combined effects of emissions from agriculture, industry, and transport, as well as concentrated urban household emission sources on exposure of the human population to AAP remain largely unknown. In addition, it is not well established how atmospheric *N*_*r*_ losses compare to losses to aquatic systems, which has implications for the management of *N*_*r*_ use and potential interventions to reduce *N*_*r*_ loss in Australia. In this paper, we compile refined N emission inventories in Australia during 1961-2013 and estimate the related health costs. Based on those datasets, we aim to develop improved understanding of (i) how the warming climate, in combination with the spatial structure of human activities, affects N losses in Australia; (ii) how Australia can address these *N*_*r*_ losses and associated health effects and costs under projected continued economic growth; and (iii) what is the most promising pathway for sustainable development under warming climate regarding N management?

## 2. Results and Discussion

### 2.1. Hotspots of N Emission to the Air

**Figure 1:**
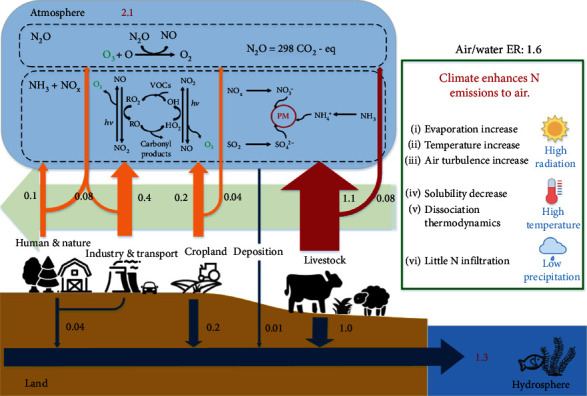
*N*_*r*_ emission to air as compared to water in Australia. ER: emission ratio. Units of these numbers are Tg N yr^−1^. The green box indicates that climate in Australia enhances N emissions to air other than water. “Livestock” includes both grazing and feedlot emissions; “Deposition” includes both dry and wet N deposition to fresh surface water; “Cropland” includes both dryland and irrigated land emissions; “Industry & Transport” includes both industry and transport emissions; “Human & Nature” includes all emissions from human, aquaculture, forest, pets, urban green-land, and waste treatment sectors. Livestock is the largest emission source, with 1.2 Tg *N*_*r*_ release to the atmosphere per year and 1.0 Tg *N*_*r*_ emissions to the hydrosphere per year. Climate in Australia enhances N emissions to air other than to water. High solar radiation increases water evaporation, surface temperature, and air turbulence, all promoting N emissions to air; high temperature increases NH_3_ volatilization rate because of its solubility and dissociation thermodynamics; low precipitation indicates little N infiltration, reducing N emissions to water.

Australia emitted an annual total of 2.1 Tg *N*_*r*_ to the air in 2013, compared to only 1.3 Tg total N (TN) released to water bodies (Figure 1). Total NH_3_, NO_x_, and N_2_O emissions were estimated at 1,350 ± 229 (mean ± standard error), 493 ± 79, and 215 ± 34 Gg N yr^−1^, respectively (Supplementary Material, Figure S1). The largest NH_3_ emission source was livestock production (1,037 ± 198 Gg N yr^−1^), including both feedlots and grasslands. Industry and transport (412 ± 56 Gg N yr^−1^) were the main sources of NO_x_ emissions, mainly from fossil fuel combustion (409 ± 55 Gg N yr^−1^). Forest emissions (79 ± 14 Gg N yr^−1^), livestock excretion (70 ± 15 Gg N yr^−1^), and agricultural soils (54 ± 4 Gg N yr^−1^) accounted for 94% of the total N_2_O emission. Grassland (1,017 Gg N yr^−1^) and cropland (212 Gg N yr^−1^) dominated the *N*_*r*_ emission to aquatic systems.

**Figure 2:**
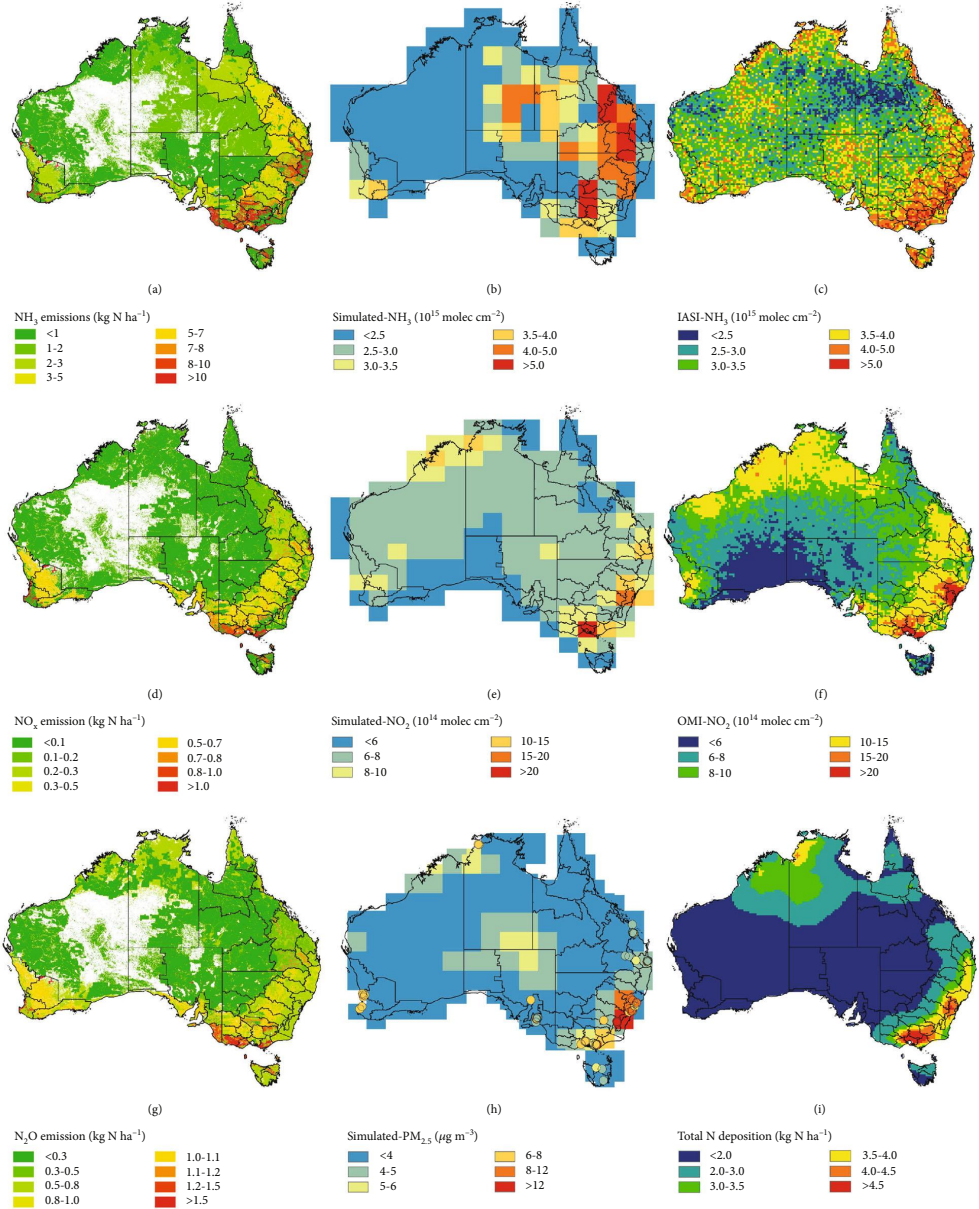
Spatial patterns of *N*_*r*_ emissions to the air across Australia in 2013 and validation: (a) NH_3_ emission; (b) simulated tropospheric NH_3_ column; (c) satellite observation of NH_3_ column; (d) NO_x_ emission; (e) simulated tropospheric NO_2_ column; (f) satellite observation of NO_2_ column; (g) N_2_O emission; (h) simulated ground-level PM_2.5_ concentrations; (i) simulated N deposition. The colored dots in (h) represent the ground-level monitoring results of PM_2.5_. Simulated NH_3_, NO_2_, and PM_2.5_ concentrations were based on *N*_*r*_ emission data from the CHANS, which further input to the GEOS-Chem atmospheric chemistry transport model for simulation. N deposition rates were simulated by satellite columns combining the vertical profiles from MOZART-4 (Model for Ozone and Related chemical Tracers, version 4) and mixed effect models based on NO_2_, NH_3_ columns, and meteorological factors. The basic map of Australia is adopted from Natural Earth (https://www.naturalearthdata.com/).

Figures 2(a), 2(d), and 2(g) show the spatial distribution of atmospheric *N*_*r*_ emissions across Australia in 2013. The greatest emission intensities of NH_3_ occurred in areas with feedlots (825 kg N ha^−1^ yr^−1^) and across the southeastern intensively managed grasslands, especially in Victoria. Relatively high N fertilization rates in these grassland areas increased N loss through NH_3_ emissions [18]. Urban areas had the largest emission intensities of NO_x_ (86 kg N ha^−1^ yr^−1^). The metropolitan areas such as Sydney and Melbourne were hotspots of NO_x_ emissions due to agglomerations of intensive industrial facilities and traffic networks. Feedlot areas had the largest emission intensity of N_2_O (59 kg N ha^−1^ yr^−1^).

To validate emission estimates and their spatial distribution, annual mean tropospheric NH_3_, NO_x_ columns, and ground-level PM_2.5_ concentrations were simulated with the GEOS-Chem atmospheric chemistry transport model based on *N*_*r*_ emission intensities (Figures 2(b), 2(e), and 2(h)) and compared with satellite observations, ground-level monitoring, and N deposition patterns (Figures 2(c), 2(f), and 2(i)). Results showed that both simulated NH_3_ and NO_2_ columns agree well with satellite observations in magnitude (quantitative comparisons in Figure S6), although the simulated NH_3_ column presents a wide range of high concentrations among the eastern Australia, while satellite observation mainly showed hotspots in southeast Australia. The difference could originate from the uncertainty of NH_3_ emission intensities, the low spatial resolution of the simulation results, or satellite observation bias in connection with NH_3_ and NH_4_^+^ aerosols, including the influence of clouds and sulfur dioxide (SO_2_) concentrations. Simulated ground-level PM_2.5_ concentrations also agreed well with ground monitoring results (Figure 2(h)) and N deposition patterns, with hotspots shown in large cities like Sydney and Melbourne. These results provide confidence in the accuracy of the emission inventories used in this study.

### 2.2. Effects of Warming Climate on Emissions

Total NH_3_ emissions for Australia have been underestimated by previous studies [16, 19, 20], mainly because the differences in emission factors due to climate trend were not adequately accounted for, and some important emission sources were not included (Supplementary Material, Section 1). Relatively lower emission factors (EFs) for NH_3_ were commonly adopted, and the EFs applied to both grazing and feedlot livestock production were the same. Previous studies also underestimated N_2_O emissions, mainly because of neglecting emissions from natural sources, e.g., emissions from forest soils.

**Figure 3:**
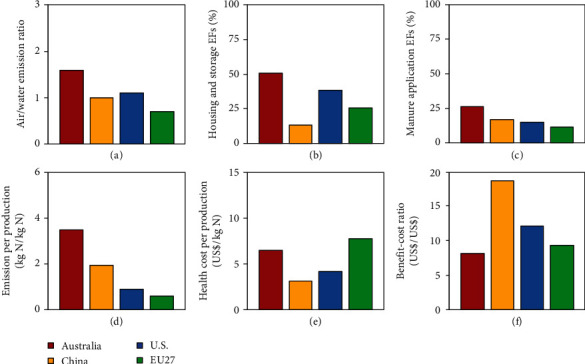
Comparison of *N*_*r*_ emission factors and associated cost and benefit between Australia and other countries: (a) air/water emission ratio; (b) livestock housing and manure storage NH_3_ emission factors; (c) manure application NH_3_ emission factors; (d) atmospheric emission per livestock production; (e) health cost per livestock production; (f) benefit-cost ratio of livestock industry (including feedlot and animal grazing). “Emission per production” represents the atmospheric N emission for each kg N accumulated in livestock products; “Health cost per production” indicates the health cost related to atmospheric N emission for each kg N accumulated in livestock products; “Benefit-cost ratio” is the ratio of income and total cost (including feed and health damage) in livestock industry.

After correcting emission factors and taking into account additional sources, we found a relatively high air/water emission ratio in Australia; 1.6 times of *N*_*r*_ was emitted to air than to water (only fresh water was considered in this study). This emission ratio is considerably higher than in China, the United States, and the European Union (Figure 3(a)), mainly because of the enhanced NH_3_ emission due to the warm, dry climate, and high solar radiation in Australia (Figure 1), indicating the necessity of developing targeted N management strategies specifically adapted to Australia and other warm regions. The NH_3_ volatilization rate increased with temperature because of its solubility and dissociation thermodynamics, especially for emissions from agriculture. High temperatures can increase NH_3_ emissions from both normal and slow-release N fertilizer applications [21, 22] and also enhance emissions from manure in livestock operations (Figures 3(b) and 3(c)). NH_3_ emissions from open dairy feedlots are nearly 90% higher in summer than in winter [23]. When manure is stored, increasing temperature induces both biotic and abiotic NH_3_ emissions [24]. For grazing animals, NH_3_ emissions present a *Q*_10_ (the relative increase over a range of 10°C) of 4.7 between the warmest and coolest months [25]. In this study, NH_3_ releases from manure application in Australia, and other countries were simulated based on micrometeorological models developed by Huijsmans [26, 27]. Results indicate that due to its warm climate and high solar radiation, NH_3_ EFs from the manure application in Australia are the highest among all evaluated countries. Details can be found in Section 2 and Table S21 in Supplementary Material. A further warming climate would aggravate the situation. An increase of 1.1°C in annual mean temperature between 1961 and 2013 [28] (Figure S7), likely a manifestation of climate change, increased NH_3_ emissions from fertilizer application and livestock by about 10% and 15% during the period, respectively.

Most *N*_*r*_ lost to aquatic systems in Australia comes from agricultural land. In principle, increases in temperature will—within limits—accelerate all biological processes, both those releasing and immobilizing *N*_*r*_. Higher temperatures and air NH_3_ concentrations could also lead to high rates of N deposition to surface water. The total N deposition to surface water was estimated at about 7 Gg N per year, according to global aerosol chemistry–climate model simulation [29], accounting for a negligible part (~0.5%) of total aquatic N inputs (Figure 1). Due to a range of potential chemical and biological feedback processes, the net effects of the warm climate on *N*_*r*_ releases to aquatic systems remain uncertain, but these effects are likely minor compared to N losses to the atmosphere [30].

### 2.3. Socioeconomic Development and Sustainable N Use

**Figure 4:**
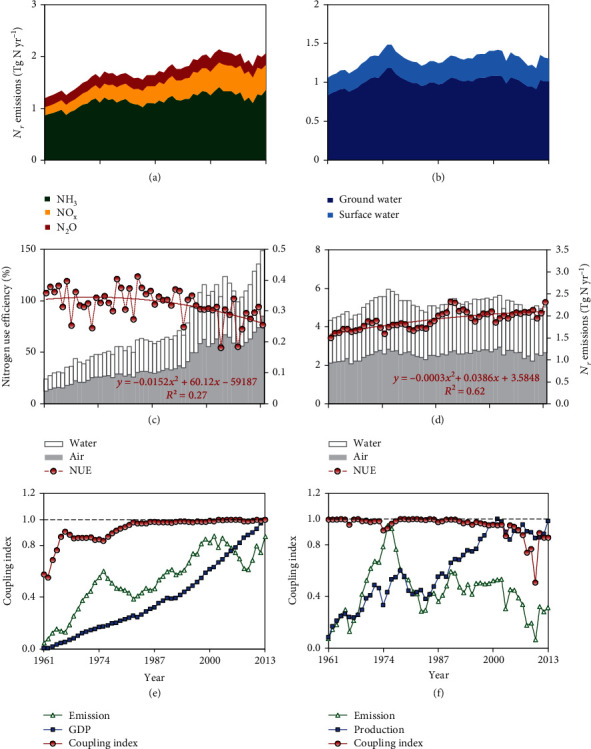
*N*_*r*_ emission inventories, N use efficiency, and coupling degree of emissions and production values in Australia during 1961-2013: (a) total *N*_*r*_ emissions to air; (b) total *N*_*r*_ emissions to water; (c) *N*_*r*_ emissions and nitrogen use efficiency (NUE) of cropland; (d) *N*_*r*_ emissions and NUE of livestock industry; (e) coupling index of total environmental *N*_*r*_ emissions with GDP; (f) coupling index of environmental *N*_*r*_ emissions with production value of livestock industry. GDP: gross domestic product. In (e, f), the green lines represent integrated environment indices, while the blue lines indicate economic growth indices. Values of all those indices only suggest the fluctuations of those two indicators on temporal scale, respectively. The red lines are coupling index (CI), expressing the degree of connection between environmental systems (*N*_*r*_ emissions as indicators) and economic systems (gross domestic production as indicators) by complex correlation model calculation. A higher CI would indicate a closer relationship between emissions and economy and less sustainable environment management, while a lower CI with low emissions would suggest decoupling and more sustainable environment management.

NH_3_ emissions in Australia increased from 0.9 ± 0.2 Tg N yr^−1^ to 1.35 ± 0.2 Tg N yr^−1^ during 1961-2013, showing a peak at 1.4 ± 0.2 Tg N yr^−1^ in 2002 (Figure 4(a)). Grazing livestock dominated NH_3_ emissions, and changes of livestock numbers contributed to the annual fluctuation. Livestock number changes were due to market dynamics and extreme weather events [31, 32]. NO_x_ emissions increased steadily from 0.16 ± 0.02 Tg N yr^−1^ in 1961 to 0.52 ± 0.08 Tg N yr^−1^ in 2007 and then slightly decreased to 0.49 ± 0.06 Tg N yr^−1^ in 2013. Industry and transport emissions dominated the trends of NO_x_ emissions. A long-term growth and a recent reduction of fossil fuel consumption contributed to the changes. N_2_O emissions increased from 0.17 ± 0.03 Tg N yr^−1^ in 1961 to 0.27 ± 0.04 Tg N yr^−1^ in 1999 and subsequently dropped to 0.22 ± 0.03 Tg N yr^−1^ in 2013. Temporal variations were mainly caused by deforestation, N deposition rates, and emissions from grazing. TN emissions to surface water increased from 0.22 Tg N yr^−1^ to 0.29 Tg N yr^−1^, with a maximum of 0.33 Tg N yr^−1^ in 2001, while TN emissions to groundwater rose from 0.84 Tg N yr^−1^ to 1.01 Tg N yr^−1^ during the period, reaching a peak at 1.18 Tg N yr^−1^ in 1976 (Figure 4(b)).

*N*_*r*_ emissions from croplands rose consistently from 0.08 Tg N yr^−1^ to 0.50 Tg N yr^−1^ during 1961-2013 (Figure 4(c)). Growth in the use of mineral fertilizer was the major cause of an increase in atmospheric *N*_*r*_ emissions, while the increasing amount of *N*_*r*_ lost to water was caused by various water emission sources, e.g., surface runoff and soil leaching of fertilizer *N*_*r*_ directly to water bodies and biological N fixation (BNF). Cropland nitrogen use efficiency (NUE) is defined as the ratio between harvested N (including crop products and removed straw) and the input N on cropland (including fertilizer, manure, BNF, and N deposition) [33]. NUE increased followed by a decreasing trend over the period between 1961 and 2013. NUE was higher than 100% during the first 30 years, indicating that arable agriculture was in fact mining soil N, which carries risk of soil degradation and desertification [19]. In Australia, the wet season in winter enhances soil mineralization. A soil mineral N test prior to fertilization in spring then finds sufficient N in soil, giving an N fertilizer application rate suggestion, which is in fact insufficient. This suggestion to farmers fails to account for dynamic balances of soil N input/output and organic/mineral N, resulting in a suggested fertilizer application rate which is too low [34]. Although Australia has improved soil testing and increased fertilizer application over time, the N inputs did yet not compensate for the N removed in grains, and soil N mining has become an ongoing problem for cropping system in recent decades, especially for wheat [35].

For livestock production, total *N*_*r*_ emission increased from 1.90 Tg N yr^−1^ to 2.20 Tg N yr^−1^ during the period, with a peak of 2.61 Tg N yr^−1^ in 1975, half of which constituted emissions to air (Figure 4(d)). Change in livestock numbers were the major cause of fluctuation in *N*_*r*_ emissions. Livestock NUE is defined as the ratio of removed N (including all animal products), and the input N (including animal feed, and grassland fertilizer, BNF, and N deposition). It steadily increased from 3.4% in 1961 to about 5.3% in 2013, mainly due to increasing grazing stock densities. Average grazing densities in pastoral zones of Australia reached 0.26 dry sheep equivalent (DSE, a standard unit denoting a two-year-old, 45 kg Merino sheep) per ha in 2011, representing a 50% growth over the previous 20 years [36]. This could be explained by shrinking grassland areas, increased supplementary feeding, increased use of legumes, and improvements in grazing management [11, 36]. Higher livestock NUE indicates smaller environmental emissions per production unit, from 7.9 kg N/kg N in 1961 to 6.6 kg N/kg N in 2013.

In order to take a quantitative approach to assessing the sustainability of Australian *N*_*r*_ use, we introduce the concept of coupling index (CI) [37], which expresses the degree of connection between environmental systems (*N*_*r*_ emissions as indicators) and economic systems (gross production values as indicators) by complex correlation model calculation. A higher CI would indicate a closer relationship between emissions and economy, while a lower CI with low emissions would suggest decoupling and more sustainable environment management. As an interpretation of our CI calculations, *N*_*r*_ emissions appear to still be increasingly coupled with GDP in Australia over time (Figure 4(e)). The trend of CI was mainly caused by the increasing use of fossil fuel and livestock number changes. Unlike CO_2_ emissions [38], a turning point for *N*_*r*_ emissions—the beginning of decoupling of emissions and economic growth—has not been observed yet for Australia. A turning point for a decoupling of NO_x_ emissions from economic development has been observed in many countries, including the USA, Germany, and Japan [39], and similarly for nitrate concentrations in groundwater, e.g., in Denmark [40]. Based on our analysis, these findings suggest that environmental N pollution and economic development are not mutually exclusive objectives for Australia in the future with a wider adoption of more sustainable management practices.

The gross economic value of livestock production increased while associated *N*_*r*_ emission fluctuated without an overall trend during the study period (Figure 4(f)). The CI generally decreased, and livestock NUE increased (Figure 4(d)), indicating an increasing efficiency of N use in livestock production. A dip of CI was found in 1974 and was caused by a fall of livestock value owing to credit and interest changes [41]; while the production value fell, emissions kept rising owing to an increase in livestock numbers. From 1990 onwards, the CI decreased to a minimum in 2010. In 2010, emissions from both sheep and livestock were low, owing to a gradual change from sheep grazing towards cattle and grain farming, originating from the policy responding to falling price of wool [31]; while the livestock production value did not decline sharply on account of the growth of meat prices [9]. In conclusion, trends of CI show that livestock production development could be combined with environmental protection in Australia. As one of the world's leading countries in livestock husbandry, if Australia was to aim towards growth of GDP and reduction of *N*_*r*_ emission and a more sustainable and efficient livestock industry, this could best be achieved by shifting focus to less-quantity, higher-quality farming.

### 2.4. Health Damage Costs

NH_3_ and NO_x_ emissions to air are the main precursors of PM_2.5_, while N_2_O emission leads to stratospheric O_3_ depletion and global warming (100 Tg CO_2_-eq per year in Australia), causing consequential health problems.

**Figure 5:**
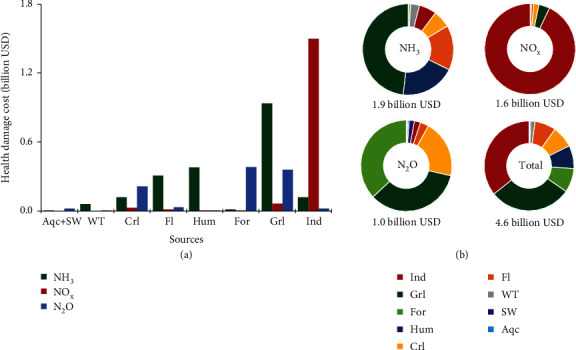
Health damage costs of atmospheric *N*_*r*_ emissions in Australia in 2013: (a) costs from emission sources; (b) cost apportionments for NH_3_, NO_x_, N_2_O, and total. Values in constant 2004-2006 USD. Aqc: aquaculture; SW: surface water; WT: waste treatment (wastewater and solid waste treatment); Crl: cropland; Hum: human; For: forest; Fl: feedlot; Grl: grassland; Ind: industry and transport. Pets and urban green-land emissions are included in the human sector.

In 2013, the health damage costs associated with AAP from total *N*_*r*_ emissions amounted to 4.6 billion USD, equivalent to 0.3% of GDP (Figure 5). Our results indicate health cost related to exposure to PM_2.5_ at 3.5 billion USD, which is in the same range as estimates by the World Bank (3.4 billion USD) [42]. Total health damage costs for Australia were quite small compared with the cost in the EU27 (100 billion € [43]) and the USA (50 billion USD [44]). The unit health damage costs varied widely in 58 regions in Australia, but most were lower than 1 USD per kg N emitted (Table S22), as compared to 2-30 € per kg N in EU27 [43] and 5-24 USD per kg N in USA [44]. Our results indicate that in most areas of Australia, *N*_*r*_ emissions led to comparatively low ambient air pollution and health damage, but in and near large cities (like Sydney and Melbourne), AAP from *N*_*r*_ emissions contributed to substantial health risks, owing to the high population density and a high rate of anthropogenic emissions (e.g., combustion of fossil fuels in industry and transport). Although the largest source of *N*_*r*_ emissions is grassland, this resulted in lower health costs than emissions from industry and transport, as grassland sources are more distant from population concentrations, and have lower damage costs per unit of *N*_*r*_ emissions. In 2013, health costs due to NH_3_ emissions are estimated at 1.9 billion USD, with a relative contribution by grassland of 48%. NO_x_ emissions caused health costs of 1.6 billion USD, with 93% of emissions stemming from fossil fuel combustion in industry and transport. Emissions of N_2_O caused health damage costs of 1.0 billion USD. Industry and transport (1.6 billion USD), grassland and grazing animals (1.4 billion USD), and forest (0.4 billion USD) were the three main contributors to the total health cost attributed to atmospheric *N*_*r*_ emissions.

Being one of the world's leading commercial livestock producers, it is essential for Australia to keep improving meat and wool quality and the productivity of its herds [9]. However, taking into account the 1.8 billion USD societal cost of AAP caused by livestock *N*_*r*_ emissions, livestock production industry (including feedlot and animal grazing) in Australia only achieved a net societal benefit of 12 billion USD in 2013, resulting in a profit margin of 87.6%, less efficient than that in the USA (91.7%) and EU27 (89.2%) (Table S24). For each kg N accumulated in livestock products, 3.5 kg *N*_*r*_ was emitted to the atmosphere in Australia, causing about 6.5 USD of health damage; while in the USA and EU27, only 0.9 and 0.6 kg *N*_*r*_ was emitted for each kg N accumulated, leading to 4.1 and 7.8 USD health cost, respectively. For each USD in total cost (including feed and health damage), livestock industry in Australia earned just 8 USD gross production value, much lower than the USA (12.0 USD) and EU27 (9.3 USD) (Figures 3(d)–3(f)). Official institutions (e.g., Meat & Livestock Australia) are providing instructions and technologies for farmers to improve the efficiency and cost effectiveness of livestock production systems.

## 3. Conclusions and Perspectives

A warming climate in Australia enhances *N*_*r*_ emissions to air relative to emissions to water, causing comparatively low N use efficiencies and atmospheric *N*_*r*_ pollution, with total health costs amounting to about 4.6 billion US dollars per year. Future climate change, with increasing average temperatures, could aggravate this situation, increasing *N*_*r*_ emissions to air in world regions which get warmer as a result. New mitigation measures and land use strategies are needed, especially for agriculture. Climate-smart agriculture could become a new management model for the Australian livestock industry, e.g., improving animal feed and waste management to reduce emissions [45]. The results of a meta-analysis study indicate that combining low crude protein diet, urease inhibitors additive for manure on the lot, and compost additive, NH_3_ emissions from beef feedlot system could be suppressed in all processes (including manure in feedlot, compost, and land application), resulting in a total decrease of 56.8% NH_3_ emissions compared to traditional systems [46]. For cropland, precision farming with optimization of input applications, including N fertilizer, could significantly reduce soil N mining (e.g., in dryland wheat) and N pollution (e.g., in vegetables and sugarcane) at the same time, allowing climate adaptation and reducing greenhouse gas emissions [47].

In recent decades, the coupling between economic growth and total *N*_*r*_ emission is gradually tightening. In 2011, the Australian Government established the “Emissions Reduction Fund” (ERF) for businesses, farmers, and land managers, to adopt smarter practices and technologies that cut the emission of greenhouse gases they create [11]. Similar approaches are required with a specific focus on *N*_*r*_ emissions, which could benefit not only the reduction of N_2_O emission in terms of climate change but also human health and ecosystem degradation, e.g., by reducing the N load to the Great Barrier Reef [48]. In view of its access to advanced production and environmental technology, Australian policies to reduce N pollution could consider moving towards more information-intensive industries and services, increased environmental awareness, enforcement of environmental regulation, better technology, and a higher level of environmental expenditure in the future.

## 4. Materials and Methods

### 4.1. Model and Dataset

The study area of this paper covers the entire terrestrial territory of Australia. We used the Coupled Human And Natural Systems (CHANS) model to evaluate the annual N fluxes in Australia during 1961-2013. The whole country was classified as 14 subsystems, including cropland, grassland, feedlot, human, industry, aquaculture, forest, pets, urban green-land, solid waste, wastewater, atmosphere, surface water, and groundwater. Inputs, outputs, and accumulation of N in each subsystem were calculated based on a mass balance approach. In the vertical direction, N deposition on land was considered as input to the system. Here, we focused on the atmosphere subsystem to identify *N*_*r*_ emissions from all other subsystems to air. A detailed description of the CHANS model can be found in Figure S1 and Gu et al. [49].

Data adopted in this study were divided into two parts: (i) information and activity data in Australia, including population, N fertilizer application, crop/livestock production, land use, and energy consumption, all derived from global statistics websites, e.g., FAO [10], IFA [8]), and the Australian Bureau of Statistics [35]; and (ii) diverse parameters (e.g., N content in crops) and emission factors (EFs) for various sources, obtained from the literature and previous studies (Table S1-S11).

Climatological effects were also considered. Volatilization of NH_3_ is highly temperature dependent, affecting the variability of NH_3_ emissions and therefore varies considerably across Australia, especially for agricultural sources [50]. We modified EFs for NH_3_ emissions accounting for the effect of climate using a climate-dependent paradigm developed by Sutton et al. [25]. Average *Q*_10_ (the relative NH_3_ volatilization increase over a range of 10°C) of 2, 2.5, and 1.25 were used for fertilizer application, excretion of cattle and horses, and excretion for other livestock, respectively. Calibration coefficients (indicators representing the impacts of annual temperature on annual total NH_3_ emissions) were calculated based on annual temperature and for yearly averages during 1961-2013 in Australia. The paradigm highlighted the interannual variation during the period. Calculation details can be found in Zhang et al. [29].

The N deposition rates in Australia were simulated by the global aerosol chemistry-climate model LMDZ-INCA, which couples the LMDZ (Laboratoire de Météorologie Dynamique, version 4) general circulation model and the INCA (INteraction with Chemistry and Aerosols, version 4) aerosol module, developed by Wang et al. (2017) [51]. Results were validated by comparison with dry N deposition from satellite columns combining the vertical profiles from MOZART-4 (Model for Ozone and Related chemical Tracers, version 4) and the wet N deposition estimated by mixed effect models based on NO_2_, NH_3_ columns, and meteorological factors [52, 53].

### 4.2. Spatial Distribution and Validation

The spatial patterns of NH_3_, NO_x_, and N_2_O emissions in Australia in 2013 were estimated. We introduced the parameter “emission intensity [15] (kg N ha^−1^ yr^−1^)” to describe the rate of emissions across all regions of the country. Emissions originating from each subsystem were quantified for corresponding regions on the Australian land use map [54]. Accordingly, an *N*_*r*_ emission quantity was divided by the area of regions to calculate the emission intensity by sector. Emissions from grazing animals were allocated to pastures in 58 Natural Resource Management (NRM) regions in Australia. Emissions from all other sources were divided geographically in equal measure. Regions without one of the 14 emission subsystems, such as desert areas, were left blank. The basic map of 58 NRM regions in Australia was derived from Australian Bureau of Statistics [55].

Satellite observations were used to validate the spatial distribution of *N*_*r*_ emissions. Average vertical column densities (VCDs) in 2013 were derived from the infrared atmospheric sounding interferometer (IASI), Centre national d'études spatiales (CNES) [56, 57] for NH_3_, and Ozone Monitoring Instrument (OMI), U.S. National Aeronautics and Space Administration (NASA) [58] for NO_2_.

The GEOS-Chem atmospheric chemistry model [59] was utilized to simulate tropospheric air pollution, based on *N*_*r*_ emissions evaluated in this study. The model simulation was conducted for the year 2013 at a horizontal resolution of 2° latitude × 2.5° longitude and 47 layers in the vertical. Ground monitoring results of PM_2.5_ concentrations were derived from Environment Protection Authority (EPA) websites for each state of Australia. Details of the GEOS-Chem model setup used can be found in Section 5 in Supplementary Material.

### 4.3. Health Damage Costs

Atmospheric *N*_*r*_ emissions in Australia lead to human health costs. The respiratory and cardiac damages induced by NH_3_ and NO_x_ through PM_2.5_ were evaluated [60, 61]. For N_2_O, the health impacts from skin cancer and cataracts from stratospheric ozone depletion were assessed. We valued health impacts by estimating costs of treatment, losses in productivity, and willingness to pay (WTP) to reduce risk of premature mortality or morbidity [43, 62]. By using the distribution of *N*_*r*_ emissions, AAP, and population density in Australia, we estimated the population exposure-response to AAP (based on Gu et al. [49] and van Grinsven et al. [43]) for NH_3_-N and NO_x_-N across 58 NRM regions in Australian in 2013. The model of NH_3_ was modified from linear to exponential function, since the health cost comes to zero when there is no population. A unified price of 4 USD kg N^−1^ was used for N_2_O considering its unified global warming potential [62]. In addition to the population exposure-response [62], all prices of premature mortality were further modified according to purchasing power parity (PPP) and gross domestic product (GDP) in Australia. We assumed that the health cost was linearly related to GDP. The calculation principles are as follows:

(1) $$\begin{array}{c} P_{{\text{NH}}_{3}}\left(D\right)=0.2192D^{0.8185}, \end{array}$$

(2) $$\begin{array}{c} P_{{\text{NO}}_{\text{x}}}\left(D\right)=0.4587D^{0.6976}, \end{array}$$

(3) $$\begin{array}{c} P_{{\text{N}}_{2}\text{O}}\left(D\right)=4, \end{array}$$

(4) $$\begin{array}{c} P_{i,j}=P_{i}\left(D_{j}\right)\times {\text{ExRate}}_{\text{EU}}\times \frac{{\text{PPP}}_{\text{A},2010}}{{\text{PPP}}_{\text{EU},2010}}\times \frac{{\text{GDP}}_{\text{A},2013}}{{\text{GDP}}_{\text{A},2010}}, \end{array}$$

(5) $$\begin{array}{c} {\text{HD}}_{i}=\underset{j}{\sum }P_{i,j}\times E_{i,j}, \end{array}$$

(6) $$\begin{array}{c} {\text{HD}}_{T}=\underset{i}{\sum }{\text{HD}}_{i}, \end{array}$$

where *i* and *j* represent the gas type (NH_3_, NO_x_, and N_2_O) and NRM region in Australia, respectively; *P*_NH_3__(*D*), *P*_NO_x__(*D*), and *P*_N_2_O_(*D*) are unit cost-population density (*D*) models for each gas; *P*_*i*,*j*_ is the *N*_*r*_ price of *i* in NRM region *j* in 2013; *P*_*i*_(*D*_*j*_) is the model-calculated price of *i* in NRM region *j*; *D*_*j*_ represents population density in NRM region *j*; ExRate_EU_ is the exchange rate from USD to euro in 2010; PPP_A,2010_ and PPP_EU,2010_ stand for PPPs of Australia and EU in 2010, values in national currency units/USD [63]; GDP_A,2013_ and GDP_A,2010_ are GDPs of Australia in 2013 and 2010, respectively, values in current A$ [64]; HD_*i*_ represent the health damage cost induced by *i*; *E*_*i*,*j*_ is the emission of *i* in NRM region *j* in 2013; and HD_*T*_ is the total health damage cost from atmospheric *N*_*r*_ in 2013. The final results are presented in 2004-2006 constant US dollar.

For the Australian livestock industry, the net production value [10] represents the gross production value excluding feed cost. Net profit is calculated as the net production value minus health cost from livestock, and the profit margin represents the ratio of net profit and gross production value.

### 4.4. Socioeconomic Correlation Index

CI was used to assess the correlation of environment and economic growth. GDP was adapted as the economic indicator, while *N*_*r*_ emissions of (a) NH_3_, (b) NO_x_, (c) N_2_O, (d) TN in surface water, and (e) groundwater were used as integrated environment indicators. The raw data (emissions and GDP) were standardized (Equation (7)), weighted by an entropy method (Equations (8)–(11)), and calculated to obtain environmental and economic indices (Equations (12)–(14)). Then, a model for assessments of the coupling relationships between two systems was used to evaluate the coupling degree (Equation (15)). Detailed calculations are as follows [37]:

(7) $$\begin{array}{c} X_{ij}^{\prime }=\frac{X_{ij}-\min\left\{X_{j}\right\}}{\max\left\{X_{j}\right\}-\min\left\{X_{j}\right\}}, \end{array}$$

(8) $$\begin{array}{c} Y_{ij}=\frac{X_{ij}^{\prime }}{\sum_{i=1}^{m}X_{ij}^{\prime }}, \end{array}$$

(9) $$\begin{array}{c} e_{j}=-\frac{1}{\ln m}\;\overset{m}{\underset{i=1}{\sum }}Y_{ij}\times \ln Y_{ij}, \end{array}$$

(10) $$\begin{array}{c} d_{j}=1-e_{j}, \end{array}$$

(11) $$\begin{array}{c} w_{j}=\frac{d_{j}}{\sum_{j=1}^{n}d_{j}}, \end{array}$$

(12) $$\begin{array}{c} S_{ij}=w_{j}\times X_{ij}^{\prime }, \end{array}$$

(13) $$\begin{array}{c} S_{i\text{GDP}}=w_{\text{GDP}}\times X_{i\text{GDP}}^{\prime }, \end{array}$$

(14) $$\begin{array}{c} S_{iE}=\overset{n}{\underset{j=1}{\sum }}S_{ij}=S_{ia}+S_{ib}+S_{ic}+S_{id}+S_{ie}, \end{array}$$

(15) $$\begin{array}{c} {\text{CI}}_{i}={\left[\frac{S_{i\text{GDP}}\times S_{iE}}{{\left(\left(S_{i\text{GDP}}+S_{iE}\right)/2\right)}^{2}}\right]}^{1/2}, \end{array}$$

where *X*_*ij*_ represents the value of indicator *j* in year *i*, *X*_*ij*_′ is the standardized value, and max{*X*_*j*_}  and min{*X*_*j*_}  indicate the maximum and minimum values of indicator *j* among all years; *Y*_*ij*_ presents the proportion of the indicator *j* in year *i*; *e*_*j*_ is the information entropy of indicator *j*, while *d*_*j*_ represents the entropy redundancy; *w*_*j*_ indicates weight of the indicator; *S*_*ij*_ is the evaluation of a single indicator; *S*_*i*GDP_ presents economic growth index in year *i*; *S*_*iE*_ is the integrated environment index in year *i*; and CI_*i*_ indicates the coupling degree of economy and environment in year *i*. For livestock systems, GDP was replaced by livestock production value, and only emissions from livestock were considered.

### 4.5. Uncertainty Analysis

10,000 Monte Carlo simulations were executed to estimate the 99% confidence intervals of NH_3_, NO_x_, and N_2_O emissions in Australia during the study period. For every emission item, Coefficients of Variation (CVs, %) were applied to data involved in the calculation (including activity data, parameters, and EFs), based on data origins and properties. Details can be found in Table S12-S16 in Supplementary Material.

## Data Availability

All data, associated protocols, and materials in this paper are publicly available. Data used to evaluate the N fluxes are divided into two parts. Information and activity data are all derived from global statistics websites (e.g., FAO and IFA) and the Australian Bureau of Statistics; diverse parameters and emission factors are obtained from the literature and previous studies, as mentioned in Materials and Methods and Supplementary Material. All adopted data and resources as well as the entire atmospheric emission inventories are shown in Supplementary Material. Specific N fluxes in atmosphere and hydrosphere subsystems are available in uploaded excel file. Detailed description of protocols to calculate these N fluxes in the CHANS model is available from https://person.zju.edu.cn/en/bjgu#930811.

## Supplementary Materials

Supplementary Materials All adopted data and resources as well as the entire atmospheric emission inventories are shown in Supplementary Material. Specific N fluxes in atmosphere and hydrosphere subsystems are available in uploaded excel file. Detailed description of protocols to calculate these N fluxes in the CHANS model is available from https://person.zju.edu.cn/en/bjgu#930811.
