# Supplementary material for: The Warming Climate Aggravates Atmospheric Nitrogen Pollution in Australia
Source: Research (Wash D C). 2021 Jun 7;2021:9804583. doi: 10.34133/2021/9804583 (PMC8254137; doi:10.34133/2021/9804583)
Supplement: Supplementary Materials — All adopted data and resources as well as the entire atmospheric emission inventories are shown in Supplementary Material. Specific N fluxes in atmosphere and hydrosphere subsystems are available in uploaded excel file. Detailed description of protocols to calculate these N fluxes in the CHANS model is available from https://person.zju.edu.cn/en/bjgu#930811. [file 9804583.f1.docx]

**Supplementary Material for**

The warming climate aggravates atmospheric nitrogen pollution in Australia

Yi Sun^1^, Baojing Gu^1,2*^, Hans van Grinsven^3^, Stefan Reis^4,5^, Shu Kee Lam^2^, Xiuying Zhang^6^, Youfan Chen^7^, Feng Zhou^8^, Lin Zhang^7^, Rong Wang^8^, Deli Chen^2^, Jianming Xu^1*^.

^1^ College of Environmental and Resource Sciences, Zhejiang University, Hangzhou, 310058, P.R. China

^2^ School of Agriculture and Food, The University of Melbourne, Melbourne, Victoria 3010, Australia

^3^ PBL Netherlands Environmental Assessment Agency, PO BOX 30314, 2500 GH The Hague, The Netherlands

^4^ UK Centre for Ecology & Hydrology, Bush Estate, Penicuik, Midlothian, EH26 0QB, UK

^5^ University of Exeter Medical School, European Centre for Environment and Health, Knowledge Spa, Truro, TR1 3HD, UK

^6^ International Institute for Earth System Science, Nanjing University, Nanjing, 210023, P.R. China

^7^ Laboratory for Climate and Ocean–Atmosphere Studies, Department of Atmospheric and Oceanic Sciences, School of Physics, Peking University, Beijing 100871, P.R. China

^8^ College of Urban and Environmental Sciences, Peking University, Beijing, 100871, P.R. China

***** Baojing Gu, Jianming Xu

**Email:** [bjgu@zju.edu.cn](mailto:bjgu@zju.edu.cn); [jmxu@zju.edu.cn](mailto:jmxu@zju.edu.cn)

**Section 1 - Inventory validation.**

We compared our N_r_ emission inventories with global database estimates and other studies (Table S17). Total NH_3_ emissions have been underestimated previously, mainly because of differences in livestock emissions. Two emission inventories in Australia[1, 2] used relatively low emission factor (EF) values for NH_3_ and applied the same EF to both grazing and feedlot livestock production systems. The International Institute for Applied Systems Analysis (IIASA)[3] only considered emissions from manure storage for sheep, dairy and other cattle, excluding other processes. In this study, emissions from livestock housing and manure storage, and from manure applied to agricultural soils were quantified separately for each livestock species. Excretion fraction, average mass, age at culling, and EFs from different processes were derived from the Intergovernmental Panel on Climate Change (IPCC)[4] and literature that only applied to Australian livestock farming practices.

Total NO_x_ emission estimates from this study are close to others where the emissions are dominated by the industrial sector. Previous studies also underestimated total N_2_O emissions from Australia, especially emissions from forests. The United Nations Framework Convention on Climate Change (UNFCCC)[5] and National Carbon Accounting System (NCAS)[6] only considered emissions from land use change and forest soil management, neglecting natural forest and background emissions. Other studies ignored emissions from all forests (E.g. EDGAR[7]). In this study, we classified natural forest areas with various tree species and used EFs from on-site measurements for each forest type. Fertilizer application and biomass burning were also included as sources of emission of N_2_O. Although forests have low N_2_O emission intensities, it was the largest N_2_O emission source in Australia.

**Section 2 - Meteorological models.**

Meteorological models established by Huijsmans[8, 9]:

$$\begin{aligned} \ln{Z\left( t \right)}_{g}=a_{0, g}+a_{t,g}*\ln t+a_{1,g}*TAN+a_{2,g}*rate+a_{3,g}*wind+a_{4,g}*radiation\# \left( S1 \right) \end{aligned}$$

$$\begin{aligned} \ln{Z\left( t \right)}_{c}=a_{0,c}+{F_{c}+a}_{t,c}*\ln t+a_{1,c}*ATAN+a_{2,c}*rate+a_{3,c}*wind+a_{4,c}*temp+F_{w}*wind\#\left( S2 \right) \end{aligned}$$

Equations (S1) and (S2) represent the NH_3_ volatilization rate from manure applied to grassland and cropland, separately. $Z\left( t \right)$ (kg NH_3_-N ha^-1^ h^-1^) is the volatilization rate at time t; TAN presents the total ammoniacal nitrogen in manure at the time of application; ATAN is the actual total ammoniacal nitrogen at time t; rate is manure application rate (m^3^ ha^-1^); wind presents wind speed (m s^-1^); radiation is solar radiation (J cm^-2^ h^-1^); temp is air temperature (℃); a_t_, a_1_, a_2_, a_3_, a_4_, and F_w_ are Coefficients for surface spreading manure; a_0_ and F_c_ are constants for surface spreading manure.

**Section 3 - Water contamination.**

N_r_ emissions to water are generally low in Australia, as the comparatively dry climate leads to limited water resources and concentrating of N_r_ pollution, and consequently high levels of water contamination. Pollution from Australian land areas contributes up to 80 percent of its marine pollution and is a major threat to the long-term health of near-shore marine systems[10]. For the Great Barrier Reef (GBR), the world’s largest coral reef ecosystem, coral cover has reduced by half during the past 30 years, owing to the surge of nutrient and sediment loads from cleared, fertilized, and urbanized catchments[11]. The Australian Government has implemented the GBR Coastal Wetlands Protection Programme (GBRCWPP), valued at 8 million USD, to improve water quality and protect wetlands in the Reef catchment area[10]. However, coral cover continued to decline after the end of the programme[11]. Further observations and continuous mitigations of water pollution are needed for the sustainability of GBR.

**Section 4 - Limitations.**

Several limitations of this study should be noted. The N_r_ emission inventories compiled in this study are based on calculations of N fluxes distinguishing 14 subsystems, and the collected raw data include activity data, parameters and EFs. We assumed that EFs are constant in space and time, however, we know that in reality, emission fluxes are influenced by a range of factors. For example, feedlot excretion emissions are affected not only by animal species, but also housing structures and manure storage system types[12]; in grazed and cultivated soils, manure and fertilizer emissions vary with soil properties, fertilizer type, spreading techniques, meteorological effects, and so on[13]. In addition, we assumed that for each sector, emissions are distributed equally in corresponding land use areas, which also contributes to the uncertainty of emission spatial patterns.

Due to atmospheric diffusion, emitted N_r_ can be transported to surrounding areas, causing a gradual decrease of VCDs moving from emission sources to adjacent areas. Emissions along the coastline may be over- or under- estimated owing to the influence of wind speed, relative humidity, and other meteorological factors[14]. These effects cannot be captured in adequate detail by static emission inventories. Calculated emission intensities are based on land use maps in Australia and are thus constrained by spatial resolution of land use information. As a consequence, emission hotspots within scattered small cities with intensive NO_x_ emissions, and for sectors with highly concentrated emissions from very small areas, like feedlots and waste treatment facilities, are not detected.

The costs evaluated in this study only cover human health damage. As a result, many other environmental costs associated with N_r_ emissions and deposition are not quantified, including biodiversity loss, eutrophication, loss of recreational use, habitat decline, etc. The unit cost of ecosystem damage is one-third to half compared to those of health damage for NH_3_ and NO_x_ in Europe[15]. However, values from the literature vary widely, and the specific ecosystem damage costs for Australia is unknown. The health damage costs of NH_3_ and NO_x_ in this study are built on regression models including unit damage cost and population density established by Gu et al[16], and the liner function of NH_3_ was modified to exponential function. Precision of the model can be indicated by referring to the correlation coefficient (R^2^) between unit costs of N_r_ and population densities, and values for both NH_3_-N (R^2^ = 0.61) and NO_x_-N (R^2^ = 0.50), are significant but not very high. Therefore, the uncertainty in estimates of health damage costs is considerable.

**Section 5 – GEOS-Chem model description.**

The GEOS-Chem atmospheric chemistry model[17] was utilized to simulate the tropospheric air pollution. The model is driven by the Modern-Era Retrospective analysis for Research and Applications version 2 (MERRA-2) assimilated meteorological data obtained from the NASA Global Modeling and Assimilation Office (GMAO), and simulates a detailed tropospheric O_3_–NO_x_–VOC–halogen–aerosol chemistry[18-20]. The model simulation was conducted for the year 2013 at a horizontal resolution of 2° latitude × 2.5° longitude and 47 layers in the vertical. Global anthropogenic emissions were from the Community Emissions Data System (CEDS) inventory, and NO_x_ and NH_3_ emissions over Australia were overwritten by estimates of this study. Here we compare model simulated annual mean tropospheric NH_3_, NO_x_ columns and ground PM_2.5_ concentrations with satellite and ground measurements over Australia.

Figure S1. N_r_ fluxes and emissions to the environment across Australia in 2013 (Gg N). TN: total nitrogen.

Figure S2. Comparison of this study with other studies and the N_r_ emission sources. **a**, NH_3_ emission comparison; **b**, NH_3_ emission sources; **c**, NO_x_ emission comparison; **d**, NO_x_ emission sources; **e**, N_2_O emission comparison; **f**, N_2_O emission sources. SW: surface water; WWT: wastewater treatment; SWT: solid waste treatment; UG: urban greenland. Pets’ emissions are included in human subsystem. EDGAR: Emission Database for Global Atmospheric Research; UNFCCC: United Nations Framework Convention on Climate Change; OECD: Organization for Economic Co-operation and Development.

Figure S3. Atmospheric N_r_ emission intensities (kg N ha^-1^) in 58 NRM regions across Australia in 2013. **a**, NH_3_; **b**, NO_x_; **c**, N_2_O; **d**, Total.

Figure S4. Health damage costs of atmospheric N_r_ emissions of 58 NRM regions across Australia in 2013. **a**, NH_3_; **b**, NO_x_; **c**, N_2_O; **d**, Total.

Figure S5. N deposition rates in Australia. **a**, dry deposition; **b**, wet deposition. Dry N deposition was simulated from satellite columns combining the vertical profiles from MOZART-4 (Model for Ozone and Related chemical Tracers, version 4), and the wet N deposition was estimated by mixed effects models based on NO_2_, NH_3_ columns and meteorological factors[21, 22].

Figure S6. Correlations of simulated tropospheric NH_3_ (10^15^ molec cm^-2^) and NO_x_ (10^14^ molec cm^-2^) columns with satellite observations. **a**, comparison of satellite observation (x axis) with simulated tropospheric column (y axis) of NH_3_; **b**, comparison of satellite observation (x axis) with simulated tropospheric column (y axis) of NO_2_. Simulated NH_3_ and NO_2_ columns were based on N_r_ emission data from the CHANS, which further input to the GEOS-Chem atmospheric chemistry transport model for simulation. The model simulation ones agree well with the satellite observations. NMB: normalized mean bias.

Figure S7. Warming climate in Australia during 1961-2013. The dots represent annual surface air temperature departures during 1961-2013[23]. Zero point is the average during the period (22.0 ℃). An increase of 1.1 °C in annual average temperature has been observed during the period.

Table S1. Sources of activity data adopted in this study.

| Subsystem | Activity data | Unit | Reference |
| --- | --- | --- | --- |
| **Cropland** | Fertilizer application | Gg N yr^-1^ | IFA[24] |
|  | Cultivated areas | ha | FAO[25] |
|  | Irrigation water volume | 1000 ML yr^-1^ | ABS[26] |
|  | Crop residue harvested | kg N yr^-1^ | FAO[25] |
|  | Crop productions | t yr^-1^ | FAO[25] |
|  |  |  |  |
| **Feedlot** | Livestock feeding | 1000t yr^-1^ | FAO[25] |
|  | Livestock productions | t yr^-1^ | FAO[25] |
|  | Livestock growth quantities | head yr^-1^ | FAO[25] |
|  |  |  |  |
| **Grassland** | Fertilizer application | Gg N yr^-1^ | IFA[24] |
|  | Irrigation water volume | 1000 ML yr^-1^ | ABS[26] |
|  | Grassland areas | 1000 ha | ABS[26] |
|  |  |  |  |
| **Human** | Protein supply quantities | g capita^-1^ yr^-1^ | FAO[25] |
|  | Population | 1000 person | FAO[25] |
|  |  |  |  |
| **Industry** | Synthetic ammonia production | kt yr^-1^ | USGS[27] |
|  | Fossil fuel consumptions | PJ yr^-1^ | Australian Government[28] |
|  | Fertilizer production | t N yr^-1^ | IFA[24] |
|  | Industrial effluent | ML yr^-1^ | Australian Government |
|  |  |  |  |
| **Aquaculture** | Aquacultural productions | t yr^-1^ | FAO[29] |
|  | Cultivation area | ha | ABS[26] |
|  |  |  |  |
| **Forest** | Forest areas | 1000 ha | ABS[26], Australian Government[30] |
|  | Forestry productions | m^3^ yr^-1^ | FAO[25] |
|  | Forest burning area | ha yr^-1^ | ABS[26], Australian Government[10] |
|  |  |  |  |
| **Pet** | Pet quantities | Million head | ABS[26] |
|  |  |  |  |
| **Urban green-land** | Urban population | 1000 person | FAO[25] |
|  |  |  |  |
| **Solid waste** | Generated solid wastes | Tg yr^-1^ | ABS[25] |
|  |  |  |  |
| **Surface water** | Surface water area | km^2^ | Australian Government[30] |

Table S2. Sources of parameters adopted in this study.

| Subsystem | Parameter | Value | Unit | Reference |
| --- | --- | --- | --- | --- |
| **Cropland** | Crop N content | See reference | % | [16, 31] |
|  | Crop residue fate | See reference | % | [26] |
|  | Biological N fixation rate | See reference | kg N ha^-1^ yr^-1^ | [32] |
|  | N concentration of irrigation | See reference | mg N L^-1^ | [33-36] |
|  | N deposition rate | See reference | mg N m^-2^ yr^-1^ | [37] |
|  |  |  |  |  |
| **Feedlot** | Livestock excretion fraction | See reference | kg N (1000 kg animal mass)^-1^ day^-1^ | [4, 38] |
|  | Livestock average mass | See reference | kg | [4] |
|  | Rearing period | See reference | day | [4] |
|  |  |  |  |  |
| **Grassland** | Clover N fixation rate | 110 | kg N ha^-1^ yr^-1^ | [32] |
|  | Non-symbiotic N fixation rate | 3 | kg N ha^-1^ yr^-1^ | [32] |
|  | N concentration of irrigation | See reference | mg N L^-1^ | [33-36] |
|  | N deposition rate | See reference | mg N m^-2^ yr^-1^ | [37] |
|  |  |  |  |  |
| **Human** | Food waste | 20 | % |  |
|  | waste food fate | See reference | % | [10] |
|  | Body N content | 2.56 | % | [39] |
|  | Bio-solid fate | See reference | % | [40] |
|  |  |  |  |  |
| **Industry** | Fossil fuel N content | Coal:1.10;  Oil: 0.25;  Gas: 1.90 | % | [10, 36] |
|  |  |  |  |  |
| **Aquaculture** | Aquatic N content | See reference | % | [41] |
|  | Ratio of fertilizer to feed | 20 | % | [42] |
|  | N deposition rate | See reference | mg N m^-2^ yr^-1^ | [37] |
|  |  |  |  |  |
| **Forest** | Fertilizer application rate | Hardwood: 9.7; Softwood: 3.4 | kg N ha^-1^ | [43] |
|  | Symbiotic N fixation rate | Eucalypt: 5.5;  Acacia: 12;  Rainforest: 5.68;  Mangrove: 7.8  Other: 7.74 | kg N ha^-1^ yr^-1^ | [44-47] |
|  | Non-symbiotic N fixation rate | Eucalypt: 1.34;  Acacia: 2.4;  Callitris:0.64;  Other: 1.46 | kg N ha^-1^ yr^-1^ | [44, 45, 48] |
|  | Wood N content | 0.607 | mg g^-1^ | [49] |
|  | Wood density | 450 | kg m^-3^ | [50] |
|  | N deposition rate | See reference | mg N m^-2^ yr^-1^ | [37] |
|  |  |  |  |  |
| **Pet** | Average mass | Dog: 23.5;  Cat: 5.2 | kg | [51] |
|  | Protein requirement | Dog: 2.5;  Cat: 5 | g d^-1^ kg^-1^ |  |
|  | Excretion disposal fate | Left on green-land: 25% dog;  Compost bins: 25% dog;  Landfill: 50% dog, 100% cat | - |  |
|  |  |  |  |  |
| **Urban**  **green-land** | Urban park area | See reference | ha (1000 residents)^-1^ | [52] |
|  | Ratio of urban green-land area to park area | 6.34 | - | [53] |
|  | Urban green-land area covers | Grass: 47;  Shrub: 6;  Tree: 39;  Hard surface: 8 | % | [54] |
|  | Fertilizer application rate | 50 | kg N ha^-1^ | [55] |
|  | Non-symbiotic N fixation rate | 13 | kg N ha^-1^ yr^-1^ | [32] |
|  | N deposition rate | See reference | mg N m^-2^ yr^-1^ | [37] |
|  |  |  |  |  |
| **Solid waste** | Waste disposal rate | See reference | % | [26] |
|  | Landfill emission gas collected | See reference | % | [26] |
|  | Leachate volume | See reference | L landfill^-1^ yr^-1^ | [10] |
|  | Leachate N content | 353 | mg L^-1^ | [56] |
|  |  |  |  |  |
| **Wastewater** | influent N content | 64.4 | mg L^-1^ | [57] |
|  | Effluent N content | See reference | mg L^-1^ | [10] |
|  | Sewage sludge N content | 2.14 | % | [58] |
|  |  |  |  |  |
| **Surface water** | N deposition rate | See reference | mg N m^-2^ yr^-1^ | [37] |

Table S3. Sources and emission factors (EFs) of NH_3_-N adopted in this study.

| Subsystem | Source | Value | Unit | Reference |
| --- | --- | --- | --- | --- |
| **Cropland** | Fertilizer and N deposition | Table S7 | % | [13, 59] |
|  | Livestock manure | 30.2 | % | [13] |
|  | Crop residue burning | Table S8 | % | [60] |
|  |  |  |  |  |
| **Feedlot** | Livestock and manure storage | Table S9 | % | [4, 10, 61] |
|  |  |  |  |  |
| **Grassland** | Manure storage | 15-18 | % | [4, 61] |
|  | Manure applied on pastures | Table S10 | % | [13, 62] |
|  | Fertilizer and N deposition | 27 | % | [63] |
|  |  |  |  |  |
| **Human** | Human excretion | 20 | % |  |
|  | Blackwater disposal | 1.81 | g m^-3^ | [10] |
|  |  |  |  |  |
| **Industry** | Biomass combustion | 18.2 | % | [60] |
|  | Ammonia synthesis | 0.21 | % | [10] |
|  | Fertilizer manufacture | 4.1 | % | [25] |
|  | Fossil fuel consumption | Table S11 | kg N PJ^-1^ | [10, 64] |
|  |  |  |  |  |
| **Aquaculture** | Total N loss | 15 | % | [64] |
|  |  |  |  |  |
| **Forest** | Fertilizer-urea | 22.5 | % | [43] |
|  | Fertilizer-other | 6.5 | % | [43] |
|  | N deposition | 3 | % | [43] |
|  | Forest burning | 27.7 | kg N ha^-1^ | [65] |
|  |  |  |  |  |
| **Urban green-land** | Fertilizer and N deposition | 12.5 | % | [64] |
|  | Livestock manure | 30.2 | % | [13] |
|  |  |  |  |  |
| **Solid waste** | Landfill emission | 0.46 | kg N t^-1^ | [64] |
|  | Compost emission | 1.05 | kg N t^-1^ | [64] |
|  |  |  |  |  |
| **Wastewater** | Wastewater disposal | 2.8 | % | [10] |

Table S4. Sources and EFs of NO_x_-N adopted in this study.

| Subsystem | Source | Value | Unit | Reference |
| --- | --- | --- | --- | --- |
| **Cropland** | Fertilizer and N deposition | Table S7 | % | [66] |
|  | Livestock manure | 0.875 | % | [67] |
|  | Biological N fixation and crop residue returning | 0.875 | % | [67] |
|  | Crop residue burning | Table S8 | % | [60] |
|  |  |  |  |  |
| **Feedlot** | Livestock and manure storage | Table S9 | % | [4, 10] |
|  |  |  |  |  |
| **Grassland** | Manure storage | 0.6 | % | [4] |
|  | Manure applied on pastures | Table S10 | % | [4] |
|  | Fertilizer and N deposition | 0.7 | % | [16] |
|  |  |  |  |  |
| **Industry** | Biomass combustion | 19.1 | % | [60] |
|  | Fossil fuel consumption | Table S11 | kg N PJ^-1^ | [10] |
|  |  |  |  |  |
| **Forest** | Forest burning | 10.1 | kg N ha^-1^ | [64] |
|  |  |  |  |  |
| **Urban green-land** | Fertilizer and N deposition | 0.66 | % | [64] |
|  | Livestock manure | 1.01 | % | [64] |

Table S5. Sources and EFs of N_2_O-N adopted in this study.

| Subsystem | Source | Value | Unit | Reference |
| --- | --- | --- | --- | --- |
| **Cropland** | Fertilizer and N deposition | Table S7 | % | [66-68] |
|  | Livestock manure | 1.25 | % | [67] |
|  | Biological N fixation and crop residue returning | 1.25 | % | [67] |
|  | Crop residue burning | Table S8 | % | [60] |
|  |  |  |  |  |
| **Feedlot** | Livestock and manure storage | Table S9 | % | [4] |
|  |  |  |  |  |
| **Grassland** | Manure storage | 0.56 | % | [4] |
|  | Manure applied on pastures | Table S10 | % | [69, 70] |
|  | Fertilizer and N deposition | 1.2 | % | [63] |
|  |  |  |  |  |
| **Industry** | Biomass combustion | 0.9 | % | [60] |
|  | Fossil fuel consumption | Table S11 | kg N PJ^-1^ | [10] |
|  |  |  |  |  |
| **Aquaculture** | Total N loss | 1.25 | % | [64] |
|  |  |  |  |  |
| **Forest** | Fertilizer | 1.2 | % | [43] |
|  | Soil emission (average) | 0.45 | kg N ha^-1^ | [71-73] |
|  | Forest burning | 2.0 | kg N ha^-1^ | [65] |
|  |  |  |  |  |
| **Urban green-land** | Fertilizer and N deposition | 3 | % | [64] |
|  | Livestock manure | 1.25 | % | [67] |
|  |  |  |  |  |
| **Solid waste** | Landfill emission | 2.7 | mg N m^-2^ h^-1^ | [74] |
|  |  |  |  |  |
| **Wastewater** | Wastewater disposal | 1.25 | % | [75] |

Table S6. Sources and EFs of N_r_ emitted to hydrosphere adopted in this study.

| Subsystem | Source | Value | Unit | Reference |
| --- | --- | --- | --- | --- |
| N_r_ runoff | | | | |
| **Cropland** | Fertilizer and N deposition | Table S7 | % | [76] |
|  | Livestock manure | 3.6 | % | [76] |
|  | Biological N fixation and crop residue returning | 3.6 | % | [76] |
|  |  |  |  |  |
| **Feedlot** | Livestock and manure storage | Table S9 | % | [4] |
|  |  |  |  |  |
| **Grassland** | Manure storage | 6 | % | [4] |
|  | Manure applied on pastures | Table S10 | % | [77] |
|  | Fertilizer and N deposition | 6 | % | [16] |
|  |  |  |  |  |
| **Aquaculture** | Total N loss | 10 | % | [64] |
|  |  |  |  |  |
| **Forest** | Fertilizer and N deposition | 3.9 | % | [43] |
|  |  |  |  |  |
| **Urban green-land** | Fertilizer and N deposition | 4.63 | % | [64] |
|  | Livestock manure | 3.6 | % | [64] |
| N_r_ leaching | | | | |
| **Cropland** | Fertilizer and N deposition | Table S7 | % | [2, 78] |
|  | Livestock manure | 8.5 | % | [79] |
|  | Biological N fixation and crop residue returning | 8.5 | % | [79] |
|  |  |  |  |  |
| **Feedlot** | Livestock and manure storage | Table S9 | % | [4, 10] |
|  |  |  |  |  |
| **Grassland** | Manure storage | 9 | % | [4] |
|  | Manure applied on pastures | Table S10 | % | [79] |
|  | Fertilizer and N deposition | 8.5 | % | [16] |
|  |  |  |  |  |
| **Forest** | Fertilizer and N deposition | 2.4 | % | [43] |
|  |  |  |  |  |
| **Urban green-land** | Fertilizer and N deposition | 4.63 | % | [64] |
|  | Livestock manure | 8.5 | % | [64] |
|  |  |  |  |  |
| **Wastewater** | Human excretion | 9 | % | [10] |
|  | Industry wastewater | 9 | % | [10] |
|  | Landfill wastewater | 9 | % | [10] |

Table S7. EFs (%) of fertilizer application adopted in cropland subsystem.

| N_r_ emission | Dryland | | Irrigated land | |
| --- | --- | --- | --- | --- |
|  | North^a^ | South^b^ | North | South |
| NH_3_-N | 16.90 | 12.30 | 16.70 | 16.70 |
| NO_x_-N | 0.66 | 0.66 | 0.66 | 0.66 |
| N_2_O-N | 3.00 | 3.00 | 3.80 | 1.25 |
| N_r_ runoff | 2.96 | 2.96 | 1.03 | 1.03 |
| N_r_ leaching | 6.30 | 6.30 | 11.75 | 11.75 |
| Area (%) | 9.06 | 86.47 | 1.56 | 2.92 |

^a^ North area includes cropland in North Territory and Queensland.

^b^ South area includes cropland in Australian Capital Territory, New South Wales, Victoria, Western Australia, South Australia and Tasmania.

Table S8. EFs (%) of crop residue combustion adopted in cropland subsystem.

| N_r_ emission | Maize | Sugar | Rice | Wheat | Other |
| --- | --- | --- | --- | --- | --- |
| NH_3_-N | 9.7 | 12.3 | 45.0 | 5.9 | 18.2 |
| NO_x_-N | 27.4 | 14.3 | 11.2 | 23.5 | 19.1 |
| N_2_O-N | 1.5 | 0.7 | 0.6 | 0.9 | 0.9 |
| N_2_ | 61.4 | 72.7 | 43.2 | 69.8 | 61.8 |

Table S9. EFs (%) of livestock housing and manure storage adopted in feedlot subsystem.

| Livestock | NH_3_-N | NO_x_-N | N_2_O-N | N_r_ runoff | N_r_ leaching |
| --- | --- | --- | --- | --- | --- |
| Chickens, broilers | 39 | 1 | 2 | 4.5 | 3.5 |
| Chickens, layers | 35 | 5 | 2 | 4.5 | 3.5 |
| Ducks | 26 | 1 | 2 | 4.5 | 3.5 |
| Turkeys | 39 | 1 | 2 | 4.5 | 3.5 |
| Breeding swine | 44 | 1 | 2 | 4.5 | 8.1 |
| Marketing swine | 43 | 1 | 2 | 4.5 | 8.1 |
| Lamb | 33 | 1 | 2 | 4.5 | 3.5 |
| Beef cattle | 59 | 1 | 2 | 4.5 | 3.5 |

Table S10. EFs (%) of manure applied on pastures in grassland subsystem.

| Livestock | NH_3_-N | NO_x_-N | N_2_O-N | N_r_ runoff | N_r_ leaching |
| --- | --- | --- | --- | --- | --- |
| Sheep | 23 | 0.6 | 0.56 | 5 | 30 |
| Lamb | 23 | 0.6 | 0.56 | 5 | 30 |
| Goat | 23 | 0.6 | 0.56 | 5 | 30 |
| Horse | 23 | 0.6 | 0.6 | 5 | 30 |
| Ass | 23 | 0.6 | 0.6 | 5 | 30 |
| Beef cattle | 23 | 0.6 | 0.62 | 5 | 30 |
| Dairy cattle | 23 | 0.6 | 0.62 | 5 | 30 |

Table S11. EFs (kg N PJ^-1^) of fossil fuel consumption in industry subsystem.

| Fossil fuel | Transportation | | |  | Industrial processes | | |
| --- | --- | --- | --- | --- | --- | --- | --- |
|  | NH_3_-N | NO_x_-N | N_2_O-N |  | NH_3_-N | NO_x_-N | N_2_O-N |
| Coal | - | - | - |  | 10 | 115043 | 411 |
| Natural gas | 425 | 93724 | 616 |  | 107 | 44739 | 62 |
| Diesel | 7854 | 77391 | 1026 |  | 1976 | 31043 | 411 |
| Petrol | 7854 | 59135 | 3695 |  | 1976 | 31043 | 411 |
| LPG | 7854 | 39647 | 1437 |  | 1976 | 31043 | 411 |

Table S12. CVs (%) of selected activity data, parameters and EFs in NH_3_ emission evaluation.

| **Sources** | **Activity data** | | **Parameter** | | **Emission factor** | |
| --- | --- | --- | --- | --- | --- | --- |
|  | **Name** | **CV (%)** | **Name** | **CV (%)** | **Name** | **CV (%)** |
| **Livestock** | Livestock growth quantities | 5 | Excretion rate | 50 | Manure left in feedlot | 50 |
|  |  |  | Rearing period | 30 |  |  |
|  |  |  | Average mass | 30 |  |  |
|  | Grazing animal populations | 5 | Proportion of grazing | 20 | Manure left on grassland | 50 |
|  |  |  | Excretion rate | 50 |  |  |
|  |  |  | Rearing period | 30 |  |  |
|  |  |  | Average mass | 30 |  |  |
| **Industry** | Fossil fuel -transportation | 5 |  |  | Fossil fuel combustion | 100 |
|  | Fossil fuel –  industrial processes | 5 |  |  | Fossil fuel combustion | 100 |
|  | Bioenergy burning | 30 |  |  | Bioenergy burning | 100 |
|  | Synthetic ammonia | 50 |  |  | Production process emission | 50 |
|  | Fertilizer production | 5 |  |  | Production process emission | 50 |
| **Cropland** | Fertilizer application | 5 |  |  | Fertilizer emission | 50 |
|  | Cultivated areas | 5 | N deposition rate | 10 | Deposited  N emission | 50 |
|  | Human and livestock manure applied | 30 |  |  | Manure emission | 50 |
|  | Crop residue harvested | 5 | Crop residue fate (burning) | 30 | Burning residue emission | 50 |
| **Grassland** | Fertilizer application | 5 |  |  | Fertilizer emission | 50 |
| **Forest** | Forest plantation area | 5 | Fertilizer application rate | 10 | Fertilizer emission | 50 |
|  | Forest areas | 10 | N deposition rate | 10 | Deposited N emission | 50 |
|  | Forest burning area | 5 |  |  | Forest burning emission | 50 |
| **Urban**  **green-land** | Urban green-land area | 10 | Fertilizer application rate | 30 | Fertilizer emission | 50 |
|  | Urban green-land area | 10 | N deposition rate | 10 | Deposited N emission | 50 |
|  | Pets manure | 30 |  |  | Applied manure emission | 50 |
| **Human** | Population | 5 | N excretion rate | 20 | Excretion emission | 50 |
|  | Population | 5 | N excretion rate | 20 | Blackwater emission | 50 |
|  |  |  | Blackwater generation rate | 30 |  |  |
| **Aquaculture** | Total N loss in aquaculture subsystem | 30 |  |  | NH_3_ emission | 50 |
| **Solid waste** | Total solid waste | 30 | Landfill rate | 10 | Landfill emission | 50 |
|  | Waste composted | 30 |  |  | Compost emission | 50 |
| **Wastewater** | Wastewater generated | 30 |  |  | Wastewater emission | 50 |

Table S13. CVs (%) of selected activity data, parameters and EFs in NO_x_ emission evaluation.

| **Sources** | **Activity data** | | **Parameter** | | **Emission factor** | |
| --- | --- | --- | --- | --- | --- | --- |
|  | **Name** | **CV (%)** | **Name** | **CV (%)** | **Name** | **CV (%)** |
| **Livestock** | Livestock growth quantities | 5 | Excretion rate | 50 | Manure left in feedlot | 50 |
|  |  |  | Rearing period | 30 |  |  |
|  |  |  | Average mass | 30 |  |  |
|  | Grazing animal populations | 5 | Proportion of grazing | 20 | Manure left on grassland | 50 |
|  |  |  | Excretion rate | 50 |  |  |
|  |  |  | Rearing period | 30 |  |  |
|  |  |  | Average mass | 30 |  |  |
| **Industry** | Fossil fuel -transportation | 5 |  |  | Fossil fuel combustion | 100 |
|  | Fossil fuel –  industrial processes | 5 |  |  | Fossil fuel combustion | 100 |
|  | Bioenergy burning | 30 |  |  | Bioenergy burning | 100 |
| **Cropland** | Fertilizer application | 5 |  |  | Fertilizer emission | 50 |
|  | Cultivated areas | 5 | N deposition rate | 10 | Deposited  N emission | 50 |
|  | Irrigation water volume | 5 | Water source | 30 | Irrigation water emission | 50 |
|  |  |  | Water N content | 100 |  |  |
|  | Human and livestock manure applied | 30 |  |  | Manure emission | 50 |
|  | Biological N fixation areas | 5 | N fixation rate | 50 | N fixation emission | 50 |
|  | Crop residue harvested | 5 | Crop residue fate  (retained/applied) | 30 | Crop residue emission | 50 |
|  | Crop residue harvested | 5 | Crop residue fate (burning) | 30 | Burning residue emission | 50 |
| **Grassland** | Fertilizer application | 5 |  |  | Fertilizer emission | 50 |
| **Forest** | Forest burning area | 5 |  |  | Forest burning emission | 50 |
| **Urban green-land** | Urban green-land area | 10 | Fertilizer application rate | 30 | Fertilizer emission | 50 |
|  | Urban green-land area | 10 | N deposition rate | 10 | Deposited N emission | 50 |
|  | Pets manure | 30 |  |  | Applied manure emission | 50 |
|  | Biological N fixation areas | 10 | N fixation rate | 50 | N fixation emission | 50 |

Table S14. CVs (%) of selected activity data, parameters and EFs in N_2_O emission evaluation.

| **Sources** | **Activity data** | | **Parameter** | | **Emission factor** | |
| --- | --- | --- | --- | --- | --- | --- |
|  | **Name** | **CV (%)** | **Name** | **CV (%)** | **Name** | **CV (%)** |
| **Livestock** | Livestock growth quantities | 5 | Excretion rate | 50 | Manure left in feedlot | 50 |
|  |  |  | Rearing period | 30 |  |  |
|  |  |  | Average mass | 30 |  |  |
|  | Grazing animal populations | 5 | Proportion of grazing | 20 | Manure left on grassland | 50 |
|  |  |  | Excretion rate | 50 |  |  |
|  |  |  | Rearing period | 30 |  |  |
|  |  |  | Average mass | 30 |  |  |
| **Industry** | Fossil fuel -transportation | 5 |  |  | Fossil fuel combustion | 100 |
|  | Fossil fuel –  industrial processes | 5 |  |  | Fossil fuel combustion | 100 |
|  | Bioenergy burning | 30 |  |  | Bioenergy burning | 100 |
| **Cropland** | Fertilizer application | 5 |  |  | Fertilizer emission | 50 |
|  | Cultivated areas | 5 | N deposition rate | 10 | Deposited  N emission | 50 |
|  | Irrigation water volume | 5 | Water source | 30 | Irrigation water emission | 50 |
|  |  |  | Water N content | 100 |  |  |
|  | Human and livestock manure applied | 30 |  |  | Manure emission | 50 |
|  | Biological N fixation areas | 5 | N fixation rate | 50 | N fixation emission | 50 |
|  | Crop residue harvested | 5 | Crop residue fate  (retained/applied) | 30 | Crop residue emission | 50 |
|  | Crop residue harvested | 5 | Crop residue fate (burning) | 30 | Burning residue emission | 50 |
| **Grassland** | Fertilizer application | 5 |  |  | Fertilizer emission | 50 |
| **Forest** | Forest areas | 5 | Different species proportion | 10 | Forest soil emission | 50 |
|  | Forest burning area | 5 |  |  | Forest burning emission | 50 |
| **Urban green-land** | Urban green-land area | 10 | Fertilizer application rate | 30 | Fertilizer emission | 50 |
|  | Urban green-land area | 10 | N deposition rate | 10 | Deposited N emission | 50 |
|  | Pets manure | 30 |  |  | Applied manure emission | 50 |
|  | Biological N fixation areas | 10 | N fixation rate | 50 | N fixation emission | 50 |
| **Aquaculture** | Total N loss in aquaculture subsystem | 30 |  |  | NH_3_ emission | 50 |
| **Solid waste** | Landfill number | 10 | Average landfill area | 25 | Landfill emission | 50 |
| **Wastewater** | Wastewater disposal | 30 |  |  | Wastewater emission | 50 |
| **Surface water** | Total runoff N | 30 |  |  | Surface water emission | 50 |

Table S15. Evaluated emissions (Gg N) and 99% confidence intervals of NH_3_, NO_x_ and N_2_O during 1961-2013 in Australia.

| Year | NH_3_ | NO_x_ | N_2_O |
| --- | --- | --- | --- |
| 1961 | 866 (-16%, 17%) | 159 (-12%, 12%) | 168 (-16%, 15%) |
| 1962 | 891 (-22%, 11%) | 165 (-10%, 14%) | 171 (-13%, 18%) |
| 1963 | 909 (-10%, 24%) | 174 (-10%, 14%) | 175 (-15%, 15%) |
| 1964 | 939 (-8%, 25%) | 185 (-9%, 15%) | 179 (-17%, 13%) |
| 1965 | 975 (-15%, 18%) | 185 (-11%, 14%) | 180 (-11%, 20%) |
| 1966 | 878 (-18%, 15%) | 192 (-11%, 13%) | 179 (-14%, 17%) |
| 1967 | 934 (-23%, 11%) | 197 (-11%, 13%) | 184 (-24%, 6%) |
| 1968 | 958 (-22%, 10%) | 214 (-9%, 15%) | 190 (-24%, 7%) |
| 1969 | 1018 (-14%, 19%) | 213 (-4%, 20%) | 194 (-15%, 17%) |
| 1970 | 1067 (-18%, 15%) | 221 (-14%, 11%) | 200 (-18%, 14%) |
| 1971 | 1095 (-15%, 18%) | 232 (-14%, 11%) | 204 (-15%, 17%) |
| 1972 | 1160 (-20%, 12%) | 240 (-15%, 10%) | 208 (-18%, 14%) |
| 1973 | 1197 (-18%, 16%) | 252 (-15%, 10%) | 207 (-17%, 14%) |
| 1974 | 1114 (-11%, 22%) | 265 (-13%, 12%) | 216 (-7%, 25%) |
| 1975 | 1215 (-17%, 17%) | 270 (-14%, 11%) | 221 (-21%, 11%) |
| 1976 | 1163 (-17%, 15%) | 284 (-14%, 11%) | 223 (-21%, 11%) |
| 1977 | 1182 (-21%, 12%) | 283 (-12%, 12%) | 216 (-26%, 5%) |
| 1978 | 1114 (-19%, 13%) | 292 (-12%, 12%) | 214 (-20%, 10%) |
| 1979 | 1156 (-13%, 20%) | 298 (-10%, 14%) | 210 (-12%, 19%) |
| 1980 | 1180 (-23%, 8%) | 298 (-19%, 5%) | 207 (-16%, 16%) |
| 1981 | 1114 (-10%, 24%) | 308 (-14%, 10%) | 207 (-15%, 16%) |
| 1982 | 1077 (-21%, 12%) | 296 (-14%, 10%) | 206 (-10%, 20%) |
| 1983 | 1073 (-17%, 15%) | 310 (-9%, 15%) | 206 (-14%, 16%) |
| 1984 | 1018 (-16%, 17%) | 323 (-15%, 9%) | 209 (-15%, 15%) |
| 1985 | 1107 (-13%, 19%) | 325 (-12%, 11%) | 213 (-16%, 15%) |
| 1986 | 1100 (-13%, 22%) | 331 (-13%, 11%) | 213 (-7%, 24%) |
| 1987 | 1092 (-26%, 6%) | 341 (-16%, 8%) | 211 (-16%, 14%) |
| 1988 | 1151 (-10%, 23%) | 358 (-10%, 13%) | 213 (-15%, 15%) |
| 1989 | 1112 (-26%, 5%) | 363 (-16%, 8%) | 219 (-15%, 16%) |
| 1990 | 1198 (-17%, 16%) | 370 (-16%, 7%) | 223 (-18%, 14%) |
| 1991 | 1243 (-16%, 16%) | 385 (-9%, 15%) | 224 (-8%, 22%) |
| 1992 | 1157 (-13%, 18%) | 383 (-7%, 16%) | 223 (-18%, 13%) |
| 1993 | 1156 (-23%, 10%) | 383 (-13%, 10%) | 222 (-15%, 16%) |
| 1994 | 1183 (-17%, 17%) | 394 (-14%, 9%) | 225 (-15%, 16%) |
| 1995 | 1180 (-17%, 17%) | 409 (-10%, 13%) | 229 (-19%, 11%) |
| 1996 | 1279 (-10%, 23%) | 427 (-15%, 9%) | 237 (-12%, 19%) |
| 1997 | 1250 (-18%, 15%) | 440 (-17%, 6%) | 236 (-3%, 27%) |
| 1998 | 1328 (-19%, 12%) | 453 (-12%, 11%) | 236 (-11%, 20%) |
| 1999 | 1318 (-20%, 12%) | 458 (-10%, 14%) | 266 (-15%, 16%) |
| 2000 | 1248 (-15%, 19%) | 461 (-16%, 8%) | 246 (-19%, 11%) |
| 2001 | 1328 (-9%, 22%) | 474 (-11%, 13%) | 262 (-12%, 20%) |
| 2002 | 1406 (-30%, 2%) | 481 (-7%, 16%) | 253 (-16%, 14%) |
| 2003 | 1329 (-14%, 17%) | 519 (-6%, 17%) | 239 (-13%, 16%) |
| 2004 | 1329 (-15%, 17%) | 501 (-13%, 11%) | 248 (-19%, 12%) |
| 2005 | 1329 (-11%, 22%) | 503 (-6%, 17%) | 228 (-19%, 12%) |
| 2006 | 1262 (-16%, 16%) | 516 (-8%, 15%) | 234 (-21%, 9%) |
| 2007 | 1308 (-21%, 10%) | 521 (-15%, 8%) | 225 (-18%, 12%) |
| 2008 | 1145 (-27%, 6%) | 520 (-10%, 13%) | 208 (-15%, 16%) |
| 2009 | 1209 (-18%, 15%) | 511 (-10%, 13%) | 212 (-13%, 17%) |
| 2010 | 1099 (-18%, 14%) | 502 (-19%, 4%) | 204 (-11%, 19%) |
| 2011 | 1272 (-19%, 12%) | 515 (-8%, 14%) | 245 (-6%, 24%) |
| 2012 | 1248 (-12%, 20%) | 506 (-13%, 10%) | 233 (-7%, 23%) |
| 2013 | 1350 (-16%, 17%) | 493 (-12%, 13%) | 215 (-15%, 16%) |

Table S16. Uncertainty contribution of different sources (yearly average in 1961-2013).

| Sources | NH_3_ | NO_x_ | N_2_O |
| --- | --- | --- | --- |
| Livestock | 81.17% | 20.68% | 43.51% |
| Industry | 0.62% | 70.62% | 1.00% |
| Cropland | 2.47% | 4.21% | 5.93% |
| Grassland | 13.20% | 2.43% | 4.78% |
| Forest | 1.09% | 2.01% | 42.46% |
| Urban green-land | 0.20% | 0.05% | 0.17% |
| Human | 0.95% | - | - |
| Aquaculture | 0.06% | - | 0.04% |
| Solid waste | 0.21% | - | 0.18% |
| Wastewater | 0.03% | - | 0.07% |
| Surface water | - | - | 1.85% |

Table S17. Comparison of atmospheric N_r_ emission (Gg N yr^-1^) with other studies.

| **N_r_ emissions** | | **Studies** | | | | | | | |
| --- | --- | --- | --- | --- | --- | --- | --- | --- | --- |
|  |  | EDGAR  (2012) | IIASA  (2015) | UNFCCC  (2013) | Denmead  (1987) | Angus  (1990s) | OECD  (2013) | NCAS  (1999) | This study  (2013) |
| **NH_3_** | Livestock | 245 | 410 | - | 650 | 573 | - | - | 781 |
|  | Agricultural soilsᵃ | 276 | 124 | - | 250 | 2298 | - | - | 468 |
|  | Other | 39 | 18 | - | 990 | 0 | - | - | 101 |
|  | Total | 560 | 552 | - | 1890 | 2871 | - | - | 1350 |
| **NO_x_** | Industry | 366 | 253 | 759 | - | - | 750 | - | 412 |
|  | Livestock | 11 | - | - | - | - | - | - | 40 |
|  | Agricultural soils | 10 | - | - | - | - | - | - | 21 |
|  | other | 19 | 27 | 172 | - | - | 16 | - | 19 |
|  | Total | 406 | 280 | 931 | - | - | 766 | - | 493 |
| **N_2_O** | Forest | - | - | 1 | - | - | - | 1 | 79 |
|  | Livestock | 54 | 4 | 2 | - | - | - | 1 | 70 |
|  | Agricultural soils | 31 | 34 | 28 | - | - | - | 35 | 54 |
|  | Other | 18 | 18 | 20 | - | - | - | 24 | 12 |
|  | Total | 103 | 56 | 51 | - | - | - | 61 | 215 |

ᵃAgricultural soils = cropland soil + grassland soil.

Table S18. Comparison of N_r_ emissions to atmosphere and hydrosphere with other countries.

| **N_r_ emission (Tg N yr^-1^)** | **Australia**  **（2013）** | **U.S.** [80]  **（2002）** | **EU27**[81]  **（2000）** | **China**[82]  **（2015）** | **World**[83]  **（21^st^ c）** |
| --- | --- | --- | --- | --- | --- |
| **Atmospheric emissions** | 2.1 | 10.1 | 7.4 | 24.2 | 113 |
| NH_3_ | 1.35 | 3.1 | 3.2 | 14.8 | 60 |
| NO_x_ | 0.5 | 6.2 | 3.5 | 7.9 | 40 |
| N_2_O | 0.2 | 0.8 | 0.7 | 1.5 | 13 |
| **Hydrospheric emissions** | 1.3 | 9.0 | 10.5 | 24.3 | 74 |
| Surface water | 0.3 | 4.8 | 10.5 | - | 70 |
| Groundwater | 1.0 | 4.2 | - | - | 4 |
| **Emission ratio** | 1.6 | 1.1 | 0.7 | 1.0 | 1.5 |

Table S19. NH_3_-N EFs of input N during each process for cattle and sheep in Australia and other countries.

|  |  | Australia | U.S. [80] | EU27[81] | China[81] |
| --- | --- | --- | --- | --- | --- |
| Dairy cow | N excretion and NH_3_ EFs  (kg N capital^-1^ yr^-1^) | 80.3 | 99.2 | 105 | 74.4 |
|  | Housing and storage | 59% | 25% | 26% | 20% |
|  | Grazing storage | 18% | - | - | 19% |
|  | Applied on cropland | 30% | 17% | 18% | 27% |
|  | Applied on pasture | 23% | 6% | 6% | 7% |
| Beef cattle | N excretion and NH_3_ EFs  (kg N capital^-1^ yr^-1^) | 77.6 | 64.2 | 41 | 48.9 |
|  | Housing and storage | 59% | 20% | 26% | 20% |
|  | Grazing storage | 18% | - | - | 19% |
|  | Applied on cropland | 30% | 27% | 18% | 27% |
|  | Applied on pasture | 23% | 6% | 4% | 7% |
| Mutton sheep | N excretion and NH_3_ EFs  (kg N capital^-1^ yr^-1^) | 5.2 | 4.2 | 15.5 | 11.2 |
|  | Housing and storage | 33% | 54% | 25% | 6% |
|  | Grazing storage | 15% | - | - | 6% |
|  | Applied on cropland | 30% | 26% | 18% | 28% |
|  | Applied on pasture | 23% | 6% | 5% | 5% |
| Wool sheep | N excretion and NH_3_ EFs  (kg N capital^-1^ yr^-1^) | 9.0 | 4.2 | 15.5 | 11.2 |
|  | Housing and storage | 33% | 54% | 25% | 6% |
|  | Grazing storage | 15% | - | - | 6% |
|  | Applied on cropland | 30% | 26% | 18% | 28% |
|  | Applied on pasture | 23% | 6% | 5% | 5% |

Table S20. Meteorological factors in Australia and other countries.

| Climate factorsᵃ | | Temperature (℃) | Wind speed (m/s) | Solar radiation (J/cm^2^/h) | Precipitation  (mm) |
| --- | --- | --- | --- | --- | --- |
| Cattle | Australia | 19.2 | 1.82 | 80.8 | 686 |
|  | U.S. | 12.0 | 2.05 | 69.8 | 826 |
|  | EU27 | 9.7 | 1.60 | 50.8 | 807 |
|  | China | 11.2 | 1.73 | 57.9 | 816 |
| Sheep | Australia | 16.3 | 1.80 | 75.6 | 568 |
|  | U.S. | 11.1 | 2.07 | 72.1 | 674 |
|  | EU27 | 10.9 | 1.51 | 55.6 | 860 |
|  | China | 6.4 | 1.93 | 63.3 | 363 |

ᵃAll climate factors are based on yearly average data, weighted by animal distribution in regions (Natural Resource Management (NRM) regions in Australia, states in U.S., countries in EU27, and provinces in China).

Table S21. Simulated NH_3_-N EFs for manure application in Australia and other countries.

| Cumulative emission  (% of TN applied) ᵃ | Grasslandᵇ | | Croplandᶜ | |
| --- | --- | --- | --- | --- |
|  | Cattle | Sheep | Cattle | Sheep |
| Australia | 29.0 | 24.6 | 19.0 | 13.8 |
| U.S. | 28.9 | 23.4 | 14.2 | 11.4 |
| EU27 | 23.2 | 19.4 | 11.3 | 10.0 |
| China | 25.0 | 22.6 | 12.6 | 8.5 |

ᵃ TN: total nitrogen.

ᵇ Segmental calculation: 0-1, 1-2, 2-4, 4-8, 8-12, 12-18, 18-24 h.

ᶜ Segmental calculation: 0-1/6, 1/6-1, 1-2, 2-4, 4-8, 8-12, 12-18, 18-24 h.

Table S22. Prices of NH_3_-N and NO_x_-N (USD / kg N) in 58 NRM regions.

| NRM region | NH_3_-N | NO_x_-N | NRM region | NH_3_-N | NO_x_-N |
| --- | --- | --- | --- | --- | --- |
| ACT | 17.5 | 19.7 | North | 1.3 | 2.1 |
| Adelaide and Mount Lofty Ranges | 20.1 | 22.2 | North Central | 1.5 | 2.3 |
| Alinytjara Wilurara | 0.0 | 0.0 | North East | 1.0 | 1.7 |
| Avon | 0.2 | 0.3 | North West | 0.9 | 1.6 |
| Border Rivers Maranoa-Balonne | 0.1 | 0.3 | Northern Agricultural | 0.2 | 0.5 |
| Border Rivers-Gwydir | 0.3 | 0.6 | Northern and Yorke | 0.6 | 1.0 |
| Burdekin | 0.4 | 0.8 | Northern Gulf | 0.0 | 0.1 |
| Burnett Mary | 1.1 | 1.9 | Northern Rivers | 1.8 | 2.9 |
| Cape York | 0.0 | 0.1 | Northern Territory | 0.1 | 0.1 |
| Central West | 0.5 | 1.0 | Perth | 43.2 | 42.6 |
| Condamine | 1.3 | 2.1 | Port Phillip and Western Port | 32.2 | 33.1 |
| Cooperative Management Area | 0.1 | 0.2 | Rangelands | 0.0 | 0.1 |
| Corangamite | 4.3 | 5.9 | South | 1.7 | 2.7 |
| Desert Channels | 0.0 | 0.1 | South Australian Arid Lands | 0.0 | 0.1 |
| East Gippsland | 0.5 | 1.0 | South Australian Murray Darling Basin | 0.6 | 1.2 |
| Eyre Peninsula | 0.2 | 0.5 | South Coast | 0.2 | 0.5 |
| Fitzroy | 0.4 | 0.7 | South East | 0.6 | 1.0 |
| Glenelg Hopkins | 0.9 | 1.6 | South East Queensland | 14.9 | 17.2 |
| Goulburn Broken | 1.4 | 2.3 | South West | 1.2 | 2.0 |
| Hawkesbury-Nepean | 7.1 | 9.1 | South West Queensland | 0.0 | 0.1 |
| Hunter-Central Rivers | 4.3 | 5.9 | Southern Gulf | 0.1 | 0.1 |
| Kangaroo Island | 0.2 | 0.5 | Southern Rivers | 2.5 | 3.8 |
| Lachlan | 0.3 | 0.6 | Southern Rivers (Jervis Bay) | 1.7 | 2.7 |
| Lower Murray Darling | 0.1 | 0.3 | Sydney Metro | 128.8 | 107.9 |
| Mackay Whitsunday | 2.6 | 3.9 | Torres Strait | 1.5 | 2.3 |
| Mallee | 0.4 | 0.8 | West Gippsland | 1.8 | 2.8 |
| Murray | 0.6 | 1.1 | Western | 0.0 | 0.1 |
| Murrumbidgee | 0.6 | 1.2 | Wet Tropics | 1.7 | 2.7 |
| Namoi | 0.5 | 0.9 | Wimmera | 0.4 | 0.8 |

Table S23. Comparison of livestock production budget with other countries.

|  | **Australia**  **(2013)** | **U.S.**  **(2002)** | **EU27**  **(2000)** | **China**  **(2015)** | **Worldᵃ**  **(2000s)** |
| --- | --- | --- | --- | --- | --- |
| **Net production valueᵇᶜ** | 14 | 103 | 156 | 175 | 682 |
| **Health damage costᶜ** |  |  |  |  |  |
| NH_3_ | 1.3 | 8 | 18 | 8.2 | 130 |
| NO_x_ | 0.08 | - | 0.6 | 0.2 | - |
| N_2_O | 0.4 | 0.04 | 0.1 | 0.03 | 3 |
| Total | 2 | 8 | 19 | 8.4 | 133 |
| **Net profitᶜᵈ** | 12 | 95 | 137 | 167 | 549 |

ªEmissions of NH_3_ and N_2_O are in 2000 and 2010, respectively.

ᵇNet production value = gross production value – cost of feed. (FAO)

ᶜValues in constant 2004-2006 billion USD.

ᵈNet profit = net production value - health damage cost.

Table S24. Comparison of profit margin of livestock production with other countries.

|  | | **Australia**  **(2013)** | **U.S.**  **(2002)** | **EU27**  **(2000)** | **China**  **(2015)** | **Worldᵃ**  **(2000s)** |
| --- | --- | --- | --- | --- | --- | --- |
|  | **Livestock production emission (g N / USD) ᵇᶜ** | | | | | |
| NH_3_ | | 71.8 | 15.5 | 9.2 | 28.6 | 30.9 |
| NO_x_ | | 2.9 | - | 0.2 | 0.4 | - |
| N_2_O | | 5.1 | 0.3 | 0.3 | 0.6 | 2.8 |
|  | **Livestock production health damage cost (USD / USD) ᵇᵈ** | | | | | |
| NH_3_ | | 0.09 | 0.07 | 0.1 | 0.05 | 0.2 |
| NO_x_ | | 0.005 | - | 0.004 | 0.001 | - |
| N_2_O | | 0.024 | 3.9e-4 | 6.4e-4 | 1.6e-4 | 0.004 |
|  | **Profit margin of livestock productionᵉ** | | | | | |
| Profit margin | | 87.6% | 91.7% | 89.2% | 94.6% | 79.3% |

ªEmissions of NH_3_ and N_2_O in world are 2000 and 2010, respectively.

ᵇValues in constant 2004-2006 USD.

ᶜLivestock production emission (g N / USD) = N_r_ emission from livestock (g N) / net livestock production value (USD).

ᵈLivestock production health damage cost (USD / USD) = livestock production emission (g N / USD) * price of N_r_ (USD / g N).

ᵉ Net livestock production value= gross production value – feed cost;

Profit margin of livestock production = (net livestock production value – total health damage cost) / gross production value * 100%;

The calculation doesn’t consider N_r_ damage to the environment or other industries.

**References:**

[1] O.T. Denmead, "An ammonia budget for Australia." Soil Res., vol. 28, no. 6, pp. 887-900, 1990.

[2] J.F. Angus, and P.R. Grace, "Nitrogen balance in Australia and nitrogen use efficiency on Australian farms." Soil Research, vol. 55, no. 6, pp. 435, 2017.

[3] "GAINS ANNEX I", International Institute for Applied Systems Analysis, accessed October 1, 2019, https://gains.iiasa.ac.at/gains/cost.ANN/index.menu?page=441&pollutant=AAP

[4] "2006 IPCC Guidelines for National Greenhouse Gas Inventories", IPCC, https://www.ipcc-nggip.iges.or.jp/public/2006gl/vol4.html

[5] "Time Series - Annex I", UNFCCC, Accessed October 1, 2018, http://di.unfccc.int/time_series

[6] R. Dalal, W. Wang, and W.J. Robertson, et al., "Emission sources of nitrous oxide from Australian agricultural and forest lands and mitigation options.". Canberra, Australia: Australian Greenhouse Office, 2003.

[7] "Emissions Data and Maps", EDGAR, https://edgar.jrc.ec.europa.eu/

[8] J.F.M. Huijsmans, J.M.G. Hol, and M.M.W.B. Hendriks, "Effect of application technique, manure characteristics, weather and field conditions on ammonia volatilization from manure applied to grassland." NJAS - Wageningen Journal of Life Sciences, vol. 49, no. 4, pp. 323-342, 2001.

[9] J.F.M. Huijsmans, J.M.G. Hol, and G.D. Vermeulen, "Effect of application method, manure characteristics, weather and field conditions on ammonia volatilization from manure applied to arable land." Atmospheric Environment, vol. 37, no. 26, pp. 3669-3680, 2003.

[10] "Australian Government-Department of the Environment and Energy", http://www.environment.gov.au/

[11] G. De'Ath, K.E. Fabricius, H. Sweatman, and M. Puotinen, "The 27-year decline of coral cover on the Great Barrier Reef and its causes." Proceedings of the National Academy of Sciences, vol. 109, no. 44, pp. 17995-17999, 2012.

[12] Z. Bai, L. Ma, and S. Jin, et al., "Nitrogen, Phosphorus, and Potassium Flows through the Manure Management Chain in China." Environmental Science & Technology, vol. 50, no. 24, pp. 13409-13418, 2016.

[13] A.F. Bouwman, L.J.M. Boumans, and N.H. Batjes, "Estimation of global NH3 volatilization loss from synthetic fertilizers and animal manure applied to arable lands and grasslands." Global Biogeochemical Cycles, vol. 16, no. 2, pp. 1-8, 2002.

[14] J. He, S. Gong, and Y. Yu, et al., "Air pollution characteristics and their relation to meteorological conditions during 2014–2015 in major Chinese cities." Environmental Pollution, vol. 223, pp. 484-496, 2017.

[15] H.J.M. Van Grinsven, M. Holland, and B.H. Jacobsen, et al., "Costs and Benefits of Nitrogen for Europe and Implications for Mitigation." Environmental Science & Technology, vol. 47, no. 8, pp. 3571-3579, 2013.

[16] B. Gu, Y. Ge, and Y. Ren, et al., "Atmospheric Reactive Nitrogen in China: Sources, Recent Trends, and Damage Costs." Environmental Science & Technology, vol. 46, no. 17, pp. 9420-9427, 2012.

[17] "GEOS-Chem model", GEOS-Chem, accessed 1 June 2020, http://www.geos-chem.org

[18] I. Bey, D.J. Jacob, and R.M. Yantosca, et al., "Global modeling of tropospheric chemistry with assimilated meteorology: Model description and evaluation." J. Geophys. Res., vol. 106, no. D19, pp. 23073-23095, 2001.

[19] X. Lu, L. Zhang, and Y. Chen, et al., "Exploring 2016–2017 surface ozone pollution over China: source contributions and meteorological influences." Atmospheric Chemistry and Physics, vol. 19, no. 12, pp. 8339-8361, 2019.

[20] J. Shao, Q. Chen, and Y. Wang, et al., "Heterogeneous sulfate aerosol formation mechanisms during winter time Chinese haze events: air quality model assessment using observations of sulfate oxygen isotopes in Beijing." Atmospheric Chemistry and Physics, vol. 19, no. 9, pp. 6107-6123, 2019.

[21] X.Y. Zhang, X.H. Lu, and L. Liu, et al., "Dry deposition of NO2 over China inferred from OMI columnar NO2 and atmospheric chemistry transport model." Atmospheric Environment, vol. 169, pp. 238-249, 2017.

[22] L. Liu, X. Zhang, and W. Xu, et al., "Estimation of monthly bulk nitrate deposition in China based on satellite NO2 measurement by the Ozone Monitoring Instrument." Remote Sensing of Environment, vol. 199, pp. 93-106, 2017.

[23] "Bureau of Meteorology", Australian Government, accessed 10 June, 2020, http://www.bom.gov.au/

[24] "IFASTAT Database", International Fertilizer Association, accessed October 18, 2019, https://www.ifastat.org

[25] "FAOSTAT Database", Food and Agriculture Organization of the United Nations, accessed 15 September 2019, http://faostat.fao.org/home/E

[26] "ABS statistics", accessed October 18, 2018, https://www.abs.gov.au/browse?opendocument&ref=topBar

[27] "United States Geological Survey-Mineral Resources Program", USGS, https://minerals.usgs.gov/

[28] "Australian Government-Department of Industry, Innovation and Science", Australian Government, accessed 18 September 2019, https://www.industry.gov.au/

[29] "Fisheries Global Information System", Food and Agriculture Organization of the United Nations, accessed 18 October 2019, http://www.fao.org/fishery/figis/en

[30] "Australian Government-Department of Agriculture and Water Resources", Australian Government, accessed 18 January 2020, http://www.agriculture.gov.au

[31] "Grain Trade Australia", GTA, https://www.graintrade.org.au/

[32] D.F. Herridge, M.B. Peoples, and R.M. Boddey, "Global inputs of biological nitrogen fixation in agricultural systems." Plant and Soil, vol. 311, no. 1, pp. 1-18, 2008.

[33] "Sydney Water", Sydney Water, http://www.sydneywater.com.au/swe/index.htm

[34] R. Congdon, and G. P. Lukacs. "Water quality aspects of irrigation run-off from the Burdekin River Irrigation Area." In *Downstream Effects of Land Use*, edited by H. A. Hunter, A. G. Eyles and G. E. Rayment. Brisbane, Australia: Queensland Department of Natural Resources, 1996.

[35] "Agriculture Victoria", Agriculture Victoria, http://agriculture.vic.gov.au/

[36] "Commonwealth Scientific and Industrial Research Organization", CSIRO, https://www.csiro.au/

[37] R. Wang, D. Goll, and Y. Balkanski, et al., "Global forest carbon uptake due to nitrogen and phosphorus deposition from 1850 to 2100." Global Change Biology, vol. 23, no. 11, pp. 4854-4872, 2017.

[38] K.A. Smith, and J.P. Frost, "Nitrogen excretion by farm livestock with respect to land spreading requirements and controlling nitrogen losses to ground and surface waters. Part 1: cattle and sheep." Bioresource Technology, vol. 71, no. 2, pp. 173-181, 2000.

[39] "Australian Government-National Health and Medical Research Council", accessed 10 July 2019, https://www.nrv.gov.au/nutrients/protein

[40] "Australian & New Zealand Biosolids Partnership", ANZBP, https://www.biosolids.com.au/

[41] S.S. De Silva, R.M. Gunasekera, and B.A. Ingram, "Performance of intensively farmed Murray cod Maccullochella peelii peelii (Mitchell) fed newly formulated vs. currently used commercial diets, and a comparison of fillet composition of farmed and wild fish." Aquaculture Research, vol. 35, no. 11, pp. 1039-1052, 2004.

[42] X. Zhang, Y. Wu, and X. Liu, et al., "Ammonia Emissions May Be Substantially Underestimated in China." Environmental Science & Technology, vol. 51, no. 21, pp. 12089-12096, 2017.

[43] B. May, P. Smethurst, and C. Carlyle, et al., "Review of fertiliser use in Australian forestry.". Melbourne, Australia: Forest & Wood Products Australia, 2009.

[44] T. S. Grove, and N. Malajczuk, "Nodule Production and Nitrogen Fixation (Acetylene Reduction) by an Understorey Legume (Bossiaea Laidlawiana) in Eucalyptus Forest." Journal of Ecology, vol. 80, pp. 303-314, 1992.

[45] H.L. Pearson, and P.M. Vitousek, "Stand dynamics, nitrogen accumulation, and symbiotic nitrogen fixation in regenerating stands of Acacia koa." Ecological Applications, vol. 11, no. 5, pp. 1381-1394, 2001.

[46] T.L. Pons, K. Perreijn, C. Van Kessel, and M.J.A. Werger, "Symbiotic nitrogen fixation in a tropical rainforest: 15N natural abundance measurements supported by experimental isotopic enrichment." New Phytologist, vol. 173, no. 1, pp. 154-167, 2007.

[47] R.Y. Lee, and S. Joye, "Seasonal patterns of nitrogen fixation and denitrification in oceanic mangrove habitats." Marine Ecology Progress Series, vol. 307, pp. 127-141, 2006.

[48] Y. Son, "Non-symbiotic nitrogen fixation in forest ecosystems." Ecological Research, vol. 16, no. 2, pp. 183-196, 2001.

[49] P. Hopmans, H.T.L. Stewart, and D.W. Flinn, "Impacts of harvesting on nutrients in a eucalypt ecosystem in southeastern Australia." Forest Ecology and Management, vol. 59, no. 1, pp. 29-51, 1993.

[50] T. Hobbs, "Review of Wood Products, Tannins and Exotic Species for Agroforestry in lower rainfall regions of southern Australia.". Canberra, Australia: Rural Industries Research and Development Corporation., 2009.

[51] "Royal Society for the Prevention of Cruelty to Animals", RSPCA, https://www.rspca.org.au/

[52] J. Byrne, N. Sipe, and G. Searle, "Green around the gills? The challenge of density for urban greenspace planning in SEQ." Australian Planner, vol. 47, no. 32010.

[53] K. Sweeney, S. Lymbery, J. Johnson, R. Smart, and G. Griffiths, "Urban Forest Strategy.". Sydney, Australia: City of Sydney, 2013.

[54] B. Jacobs, N. Mikhailovich, and C. Delaney, "Benchmarking Australia's Urban Tree Canopy: An I-Tree Assessment, Final Report.". Sydney, Australia: Institute for Sustainable Futures, 2014.

[55] L. van Delden, E. Larsen, D. Rowlings, C. Scheer, and P. Grace, "Establishing turf grass increases soil greenhouse gas emissions in peri-urban environments." Urban Ecosystems, vol. 19, no. 22016.

[56] J. Scott, D. Beydoun, R. Amal, G. Low, and J. Cattle, "Landfill Management, Leachate Generation, and Leach Testing of Solid Wastes in Australia and Overseas." Critical Reviews in Environmental Science and Technology, vol. 35, no. 3, pp. 239-332, 2005.

[57] "Information and services for South Australians", Government of South Australia, https://www.sa.gov.au/

[58] A. B. Rosenani, D. R. Kala, and C. Ishak, "Characterization of Malaysian sewage sludge and nitrogen mineralization in three soils treated with sewage sludge." Malaysian Journal of Soil Science, vol. 12, pp. 103-112, 2008.

[59] P. E. Bacon, and J. R. Freney, "Nitrogen loss from different tillage systems and the effect on cereal grain yield." Nutrient Cycling in Agroecosystems, vol. 20, no. 2, pp. 59-66, 1989.

[60] M. He, J. Zheng, S. Yin, and Y. Zhang, "Trends, temporal and spatial characteristics, and uncertainties in biomass burning emissions in the Pearl River Delta, China." Atmospheric Environment, vol. 45, no. 24, pp. 4051-4059, 2011.

[61] O.T. Denmead, D. Chen, and D.W.T. Griffith, et al., "Emissions of the indirect greenhouse gases NH3 and NOx from Australian beef cattle feedlots." Australian Journal of Experimental Agriculture, vol. 48, no. 2, pp. 213-218, 2008.

[62] P. Balsari, E. Dinuccio, E. Santoro, and F. Gioelli, "Ammonia emissions from rough cattle slurry and from derived solid and liquid fractions applied to alfalfa pasture." Australian Journal of Experimental Agriculture, vol. 48, pp. 198-201, 2008.

[63] G. Schwenke, W. Manning, and B. Haigh, "Ammonia volatilisation from nitrogen fertilisers surface-applied to bare fallows, wheat crops and perennial-grass-based pastures on Vertosols." Soil Research, vol. 52, pp. 805-821, 2014.

[64] B. Gu, X. Ju, J. Chang, Y. Ge, and P.M. Vitousek, "Integrated reactive nitrogen budgets and future trends in China." Proceedings of the National Academy of Sciences, vol. 112, no. 28, pp. 8792-8797, 2015.

[65] T.E.L. Smith, C. Paton-Walsh, and C.P. Meyer, et al., "New emission factors for Australian vegetation fires measured using open-path Fourier transform infrared spectroscopy – Part 2: Australian tropical savanna fires." Atmos. Chem. Phys., vol. 14, no. 20, pp. 11335-11352, 2014.

[66] B. Macdonald, O. Denmead, and I. White, "Quantification of NOx and NH3 emissions from two sugarcane fields." Soil Research, vol. 52, pp. 833-840, 2014.

[67] R.C. Dalal, W. Wang, G.P. Robertson, and W.J. Parton, "Nitrous oxide emission from Australian agricultural lands and mitigation options: a review." Soil Research, vol. 41, no. 2, pp. 165, 2003.

[68] P. Thorburn, J. Biggs, K. Collins, and M. E. Probert, "Using the APSIM model to estimate nitrous oxide emissions from diverse Australian sugarcane production systems." Agriculture Ecosystems & Environment, vol. 136, pp. 343-350, 2010.

[69] S. Howden, D. White, and P. J. Bowman, "Managing sheep grazing systems in southern Australia to minimise greenhouse gas emissions: Adaptation of an existing simulation model." Ecological Modelling, vol. 86, pp. 201-206, 1996.

[70] S. Saggar, N. Bolan, R. Bhandral, C.B. Hedley, and J. Luo, "A review of emissions of methane, ammonia, and nitrous oxide from animal excreta deposition and farm effluent application in grazed pastures." New Zealand Journal of Agricultural Research, vol. 47, pp. 513-544, 2004.

[71] B. Fest, S. J. Livesley, M. Drösler, E. van Gorsel, and S. Arndt, "Soil-atmosphere greenhouse gas exchange in a cool, temperate Eucalyptus delegatensis forest in south-eastern Australia." Agricultural and Forest Meteorology, vol. 149, pp. 393-406, 2009.

[72] L. Breuer, H. Papen, and K. Butterbach-Bahl, "N2O emission from tropical forest soils of Australia." Journal of Geophysical Research Atmospheres, vol. 105, pp. 353-367, 2000.

[73] J. Kreuzwieser, J. Buchholz, and H. Rennenberg, "Emission of Methane and Nitrous Oxide by Australian Mangrove Ecosystems." Plant Biology, vol. 5, no. 4, pp. 423-431, 2003.

[74] J. Rinne, M. Pihlatie, and A. Lohila, et al., "Nitrous Oxide Emissions from a Municipal Landfill." Environmental Science & Technology, vol. 39, no. 20, pp. 7790-7793, 2005.

[75] B.J. Ni, L. Ye, Y. Law, C. Byers, and Z. Yuan, "Mathematical modeling of nitrous oxide (N2O) emissions from full-scale wastewater treatment plants." Environ Sci Technol, vol. 47, no. 14, pp. 7795-7803, 2013.

[76] P.J. Thorburn, J.S. Biggs, S.J. Attard, and J. Kemei, "Environmental impacts of irrigated sugarcane production: Nitrogen lost through runoff and leaching." Agriculture, Ecosystems & Environment, vol. 144, no. 1, pp. 1-12, 2011.

[77] L. L. Burkitt, "A review of nitrogen losses due to leaching and surface runoff under intensive pasture management in Australia." Soil Research, vol. 52, pp. 621-636, 2014.

[78] P. Prasertsak, J.R. Freney, and O. Denmead, et al., "Effect of fertilizer placement on nitrogen loss from sugarcane in tropical Queensland." Nutrient Cycling in Agroecosystems, vol. 62, pp. 229-239, 2002.

[79] C. Wachendorf, F. Taube, and M. Wachendorf, "Nitrogen Leaching from 15N Labelled Cow Urine and Dung Applied to Grassland on a Sandy Soil." Nutrient Cycling in Agroecosystems, vol. 73, no. 1, pp. 89-100, 2005.

[80] Doering, OC III., J.N. Galloway, and T.L. Theis, et al., "Reactive nitrogen in the united states: An analysis of inputs, flows, consequences, and management options.". Washington, DC: US Environ. Prot. Agency Science Advisory Board Integrated Nitrogen Committee, 2011.

[81] A. Leip, B. Achermann, and G. Billen, et al. "Integrating nitrogen fluxes at the European scale." In *The European Nitrogen Assessment*, edited by Mark A. Sutton, Clare M. Howard, Jan Willem Erisman, Gilles Billen, Albert Bleeker, Peringe Grennfelt, Hans van Grinsven and Bruna Grizzetti, 345-376. Cambridge, U.K.: Cambridge University Press, 2011.

[82] B. Gu, and X. Zhang. "Reactive Nitrogen Budgets in China." In *Atmospheric Reactive Nitrogen in China*, edited by X. Liu and E. Du, 87-109. Singapore: Springer Nature, 2020.

[83] D. Fowler, M. Coyle, and U. Skiba, et al., "The global nitrogen cycle in the twenty-first century." Phil. Trans. R. Soc. B, vol. 368, no. 1621, pp. 20130164, 2013.
